# The significance of glutaredoxins for diabetes mellitus and its complications

**DOI:** 10.1016/j.redox.2024.103043

**Published:** 2024-01-20

**Authors:** Mengmeng Zhou, Eva-Maria Hanschmann, Axel Römer, Thomas Linn, Sebastian Friedrich Petry

**Affiliations:** a Clinical Research Unit, Medical Clinic and Polyclinic III, Center of Internal Medicine, Justus Liebig University, Giessen, Germany; b Experimental and Translational Research, Department of Otorhinolaryngology, University Hospital Essen, Essen, Germany

**Keywords:** Diabetes mellitus, Glutaredoxin, Beta-cell, Redox signaling, Oxidative distress

## Abstract

Diabetes mellitus is a non-communicable metabolic disease hallmarked by chronic hyperglycemia caused by beta-cell failure. Diabetic complications affect the vasculature and result in macro- and microangiopathies, which account for a significantly increased morbidity and mortality. The rising incidence and prevalence of diabetes is a major global health burden. There are no feasible strategies for beta-cell preservation available in daily clinical practice. Therefore, patients rely on antidiabetic drugs or the application of exogenous insulin. Glutaredoxins (Grxs) are ubiquitously expressed and highly conserved members of the thioredoxin family of proteins. They have specific functions in redox-mediated signal transduction, iron homeostasis and biosynthesis of iron-sulfur (FeS) proteins, and the regulation of cell proliferation, survival, and function. The involvement of Grxs in chronic diseases has been a topic of research for several decades, suggesting them as therapeutic targets. Little is known about their role in diabetes and its complications. Therefore, this review summarizes the available literature on the significance of Grxs in diabetes and its complications. In conclusion, Grxs are differentially expressed in the endocrine pancreas and in tissues affected by diabetic complications, such as the heart, the kidneys, the eye, and the vasculature. They are involved in several pathways essential for insulin signaling, metabolic inflammation, glucose and fatty acid uptake and processing, cell survival, and iron and mitochondrial metabolism. Most studies describe significant changes in glutaredoxin expression and/or activity in response to the diabetic metabolism.

In general, mitigated levels of Grxs are associated with oxidative distress, cell damage, and even cell death. The induced overexpression is considered a potential part of the cellular stress-response, counteracting oxidative distress and exerting beneficial impact on cell function such as insulin secretion, cytokine expression, and enzyme activity.

## Graphical abstract

**Figure:**
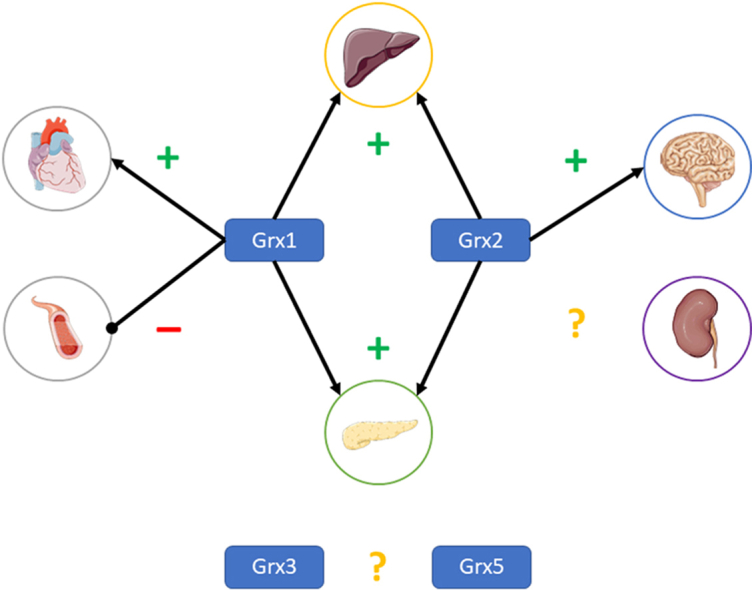

## 1 Introduction

### 1.1 Diabetes mellitus

Diabetes mellitus comprises a group of clinical syndromes characterized by chronic and persistent hyperglycemia caused by defective insulin secretion and/or insulin resistance. It has a worldwide prevalence of around 1 in 10 adults. Uncontrolled diabetes leads to long-term disturbances in carbohydrate as well as fat and protein metabolism [1]. According to the clinical manifestation, pathophysiology, and etiology, diabetes mellitus can be classified in different types. The most prevalent forms are type 1 and type 2 diabetes. In type 1 diabetes the autoimmune destruction of pancreatic beta-cells results in an absolute deficiency of insulin. Type 2 diabetes, the most prevalent form of diabetes, is caused by the interaction of varying degrees of insulin deficiency and tissue insulin resistance.

During the onset and progression of diabetes, insulin deficiency is strongly associated with diminished numbers and dysfunction of pancreatic beta-cells [2]. The unique insulin-producing cells suffer from the detrimental impact of glucotoxicity as induced by hyperglycemia [3], lipotoxicity as mediated by hyper- and dyslipidemia as well as ectopic fat storage [4], and a chronic inflammatory state [5].

Despite decades of diabetes research, no feasible strategies for beta-cell preservation are available in daily practice, indicating the necessity to identify novel therapeutic targets.

### 1.2 Redox signaling and mammalian glutaredoxins

Many clinical risk factors have been linked to promote oxidative distress, a contributing factor to the onset and/or progression of every major disease [6,7]. It was originally defined as an imbalance between oxidants and antioxidants with increased levels of reactive oxygen species (ROS) leading to irreversible oxidation of biomolecules, cell damage, and cell death. Interestingly, when thioredoxins (Trxs) and glutaredoxins (Grxs) were originally discovered, they were described as electron donors, i.e., reductants, for ribonucleotide reductase in *E. coli* [8,9]. Trxs and Grxs are often referred to as antioxidants acting in the cellular defense against oxidants. However, research of the last decades has shown that i) hydrogen peroxide (H_2_O_2_), hydrogen sulfide (H_2_S), and nitric oxide (NO) act as second messengers in redox-mediated signal transduction and that ii) Trxs and Grxs are key regulators of specific and reversible redox reactions that are linked to specific cellular functions such as proliferation, differentiation, and metabolism [[10], [11], [12]]. As such, the regulation of reversible, posttranslational Cys modifications or so-called thiol switches as part of redox signaling has been defined as oxidative eustress. The dysregulation or disruption of physiological redox signaling has been re-defined as oxidative distress (reviewed in Refs. [11,12]).

#### 1.2.1 Structure and physiological role of mammalian glutaredoxins

In the 1970s, Glutaredoxin (Grx) was discovered as glutathione (GSH)-dependent oxidoreductase, first in *E. coli* [9,13] and a few years later in calf thymus [13]. Grxs belong to the Thioredoxin (Trx) protein superfamily, which shares the classical structural motif known as the Trx fold, consisting of four-stranded β-sheets surrounded by three α-helices. They share a common active site motif Cys-X-X-Cys/Ser [14] and bind GSH [15]. Based on the number of active site cysteine residues, Grxs can be divided into the classical dithiol/class I (Cys-X-X-Cys) and monothiol/class II (Cys-X-X-Ser) proteins [16]. The latter can further be subdivided into single- and multi-domain Grxs [17]. Note, that the classification and diversity of Grxs, based on the number of active site Cys residues, but also structural differences including catalytic properties, ability to bind iron-sulfur (FeS) cofactors, and on the basis of phylogenetic analysis varies in different organisms such as bacteria, yeast, plants, and mammals [[18], [19], [20], [21], [22]]. Here, we focus on the mammalian Grxs and will exclusively use the terms dithiol and monothiol Grxs. They are ubiquitously expressed and their tissue- and cell type specific distribution has extensively been studied in mouse [23]. The human redox atlas does not contain the distribution of Grxs, however, includes glutathione reductase (GR) and γ-glutamyl cysteinyl synthase (gGCS), the rate limiting enzyme in GSH synthesis, which are important for Grx function [24].

**Fig. 1:**
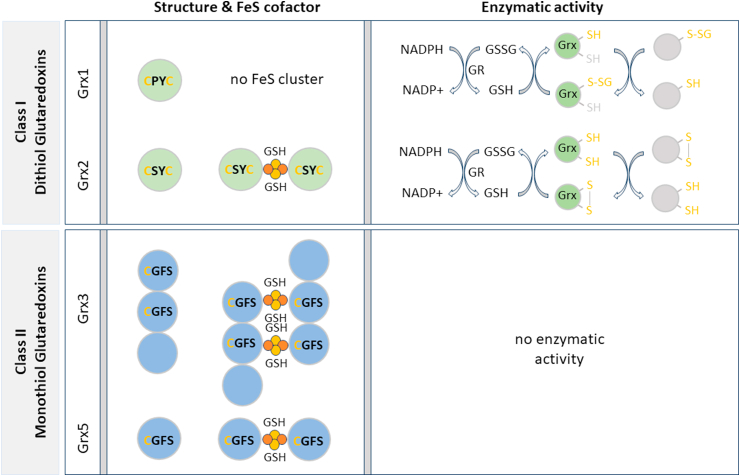
**Structure and enzymatic activity of human Grxs.** 4 Grxs have been described in humans. The dithiol Grx1 encodes the CPYC active site and does not coordinate an FeS cluster. It catalyzes the monothiol (upper reaction) and dithiol (lower reaction) mechanisms with electrons donated from NADPH and glutathione (GSH), which is reduced by glutathione reductase (GR). The dithiol Grx2 encodes the CSYC active site and coordinates a 2Fe2S cofactor. The dimeric holo-protein is enzymatically inactive. The apo-protein catalyzes monothiol and dithiol reaction mechanisms. The monothiol Grx3 contains 3 domains including two Grx domains that contain the monothiol CGFS active site. Both domains coordinate a 2Fe2S cluster and form a dimer. The monothiol Grx5 also forms a dimer. It possesses the CGFS active site motif and coordinates a 2 Fe2S cluster. Both monothiol Grxs are enzymatically inactive.

Dithiol Grxs function as oxidoreductases and donate electrons to different metabolic pathways. Two distinct but functionally related catalytic mechanisms have evolved (Fig. 1). In the dithiol mechanism or disulfide exchange reaction, Grx catalyzes the reduction of disulfide bonds within specific substrate proteins, thereby regulating protein function and participating in redox-mediated signal transduction. Briefly, the N-terminal active site Cys initiates a nucleophilic attack on the target disulfide, leading to the formation of a covalently bound mixed disulfide between Grx and the substrate (intermediate state), which is then reduced by the C-terminal active site Cys. For the reduction of Grx, electrons are delivered by NADPH via GR and 2 molecules of GSH. Under certain conditions Grx can also accept electrons from thioredoxin reductase (TrxR) [25]. In the monothiol mechanism or the de-glutathionylation reaction, the N-terminal active site Cys initiates a nucleophilic attack on the glutathione moiety of its glutathionylated substrate (substrate-S-SG). This leads to the reduction of the substrate and the formation of a mixed disulfide between Grx and GSH (Grx-S-SG), which is reduced by another GSH molecule. This process requires two distinct GSH interaction sites: one for the interaction with the substrate-S-SG and another one for the efficient reduction by GSH [26]. Grx can also catalyze the glutathionylation of proteins, depending on cellular concentration and redox potential of reduced (GSH) and oxidized (GSSG) glutathione. Upon the transient increase in GSSG, Grx catalyzes protein glutathionylation at the expense of oxidized glutathione [27].

Monothiol Grxs lack the C-terminal active site Cys and are not only incapable of catalyzing the dithiol, but also the monothiol mechanisms [16,28]. The N-terminal active site Cys residue coordinates and transfers FeS cofactors and is thereby involved in iron metabolism and homeostasis [17,[29], [30], [31]]. This can be explained by looking at structure-function relationships. A distinct loop structure adjacent to the active site and the orientation of the active site phenylalanine affect the i) orientation and reactivity of the GSH molecules and as a consequence ii) the ligation and orientation of the FeS cofactor and iii) the conformation of the holo-protein [27].

#### 1.2.2 Glutaredoxin 1

The dithiol Grx1 is located in the cytoplasm and in the mitochondrial [32] as well as nuclear intermembrane space [33]. It was also found extracellularly, indicating secretion [33]. It regulates proteins and signaling pathways involved in cell survival, death, and proliferation via reversible glutathionylation, including mitochondrial complex I [34], ASK-1/SEK-1/JNK-1 [35], NF-κB [[36], [37], [38], [39], [40]], GAPDH/Sirtuin-1 [41], Akt-FoxO [[42], [43], [44]] and protein tyrosine phosphatase 1B (PTP1B) [45]. Grx1 has been shown to attenuate apoptosis following elevated levels of H_2_O_2_ and protein carbonylation induced by high glucose in vascular endothelial cells (EC) [39,40], as well as high glucose-induced cardiac matrix metalloproteinase-induced cardiotoxicity [46], and cardiac ischemia/reperfusion injury [44]. Grx1 has been shown to be neuroprotective, whereas its upregulation in microglia promotes proinflammatory cytokine production [47]. In addition, Grx1 can maintain copper homeostasis and prevent copper-mediated oxidative damage through deglutathionylation of copper-transporting ATPases [48].

#### 1.2.3 Glutaredoxin 2

The dithiol Grx2 exhibits three splice variants in humans, namely Grx2a (mitochondrial), Grx2b, and Grx2c (nucleus and cytoplasm) [[49], [50], [51]]. It shows a 36% sequence identity with Grx1 and can be reduced by both GSH/GR and the Trx/TrxR system [52]. Grx2a is expressed in several tissues, while Grx2b and Grx2c are exclusive to testis and tumor cells [49]. Grx2 has been shown to regulate multiple signaling pathways including Ras/PI3K/Akt, JNK/AP-1, NF-κB [37], and Glycogen Synthase Kinase 3 Beta (GSK-3β) [53]. In HeLa cells, overexpression of Grx2 reduced apoptosis sensitivity [54], and its silencing had opposite effects [55]. Grx2 knockout mice develop cardiac hypertrophy, hypertension, and premature age-dependent cataracts [56,57]. In the mitochondrial Grx2-depleted mice, increased body weight, elevated plasma free fatty acid (FFA) levels, impaired hepatic glycogen synthesis, and abnormal mitochondrial structure and function were observed [58]. Overexpression of the protein protected mice against doxorubicin-induced cardiotoxicity [59], renal ischemia and reperfusion damage [60], and neuronal apoptosis [61]. Potential disulfide-substrates of Grx2 were identified in different murine tissues or human HeLa cells using the intermediate trapping approach. The identified proteins included 7 metabolic enzymes Arginase (identified in liver), GAPDH (identified in brain), Glycine N-methyltransferase (identified in liver), Inosine-5′-monophosphate dehydrogenase 2 (identified in HeLa cells), D-3-phosphoglycerate dehydrogenase (identified in HeLa cells), Enoyl-CoA hydratase (identified in liver) and UDP-N-acetyl-α-d-galactosaminyltransferase 7 (identified in testis). Using immunoprecipitation, the interaction of Grx2 and GAPDH was verified in HeLa cells, overexpressing Grx2c [62].

Mitochondria synthesize ATP via oxidative phosphorylation. Interestingly, complex I is a major source of mitochondrial ROS, and glutathionylation of complex I has been shown to inhibit its activity and increase superoxide production [63]. Grx2 regulates the mitochondrial respiratory chain by the reversible glutathionylation of complex I [64], which Beer et al. saw as a response to the oxidation of the mitochondrial GSH pool [65]. The activity of complex I in the lens of Grx2 knockout mice was only 50% of the control group with a 10% reduced ATP pool [57].

In contrast to Grx1, the active site of Grx2 is Cys-Ser-Tyr-Cys, ensuring different biochemical properties [66]. Grx2 can form 2Grx2-[2Fe–2S]-2GSH dimers. GSH is in constant exchange with the free pool of GSH. Therefore, cellular redox changes in the GSH pool regulate the oxidoreductase activity of Grx2, which has been described as a redox sensor [66,67]. Deletion of Grx2 causes a decrease in the mRNA expression of several FeS proteins serving as subunits of complex I, namely NADH dehydrogenase FeS protein 3, 7, and 8, a decline in the content of bound iron in liver mitochondria, and a corresponding increase in lipid peroxidation products [58].

#### 1.2.4 Glutaredoxin 3

The monothiol Grx3 protein is located in the cytosol under reducing conditions and translocates to the nucleus when exposed to H_2_O_2_ [[68], [69], [70]]. It is known as protein kinase C (PKC)-interacting cousin of Trx (PICOT) with the ability to interact with the protein kinase Cθ subunit (PKCθ) and PKC. Previous research has shown that Grx3 overexpression suppresses the activation of the JNK/AP-1 and NF-κB pathways in T-cells [68]. There is also evidence that Grx3 is involved in PKCθ-independent biological functions [71]. Grx3 contains three highly conserved domains, including an N-terminal Trx homology domain and two Grx homology domains, also known as PICOT homology domains, which belong to the multidomain monothiol Grx [72,73]. However, the Grx3 active site lacks a Cys and thus does not possess the classical Trx or Grx catalytic activity [16]. It was reported that Grx3 is upregulated in hypertrophic cardiomyocytes to enhance cardiomyocyte contractility and inhibit cardiac hypertrophy [[74], [75], [76]]. It plays a role in the biological process of embryogenesis [77,78] and is significantly increased in many human cancer types such as lung cancer, breast cancer, and colon cancer [[79], [80], [81]]. Nuclear-targeted Grx3 overexpression enhances the cellular resistance to diamide and mitigates thiol oxidation [70]. Grx3 knockdown results in inhibition of Ataxia telangiectasia and Rad3 related (ATR)-dependent signaling pathways that promote DNA damage repair [82]. Note that Grx3 has also been described as FeS protein. It can form a 2Grx3-2[2Fe–2S]-4GSH dimer [83], utilizing both active site Cys residues within the two monothiol Grx domains and four molecules of GSH [83]. Further research showed that in HeLa cells with Grx3 knockdown, the content of cellular iron increased and the activities of various iron-dependent proteins (cytochrome *c* oxidase, ferrochelatase, succinate dehydrogenase, and mitochondrial aconitase) decreased, suggesting that the absence of Grx3 makes the absorbed iron ineffective, indicating that Grx3 plays a central role in cellular iron homeostasis [84].

#### 1.2.5 Glutaredoxin 5

The biogenesis of cellular FeS proteins is the most basic and minimal function of mitochondria [85]. Grx5 is a mitochondrial single-domain monothiol FeS protein [30,[86], [87], [88]] and central part of the complex and highly conserved mitochondrial FeS cluster assembly machinery (reviewed in e.g. Refs. [89,90]). Briefly, Grx5 acts as an intermediate FeS cluster carrier. It receives *de novo* synthesized [2Fe–2S] clusters from scaffold proteins and transfers them to target apoproteins, a process which is well-orchestrated by a myriad of factors [85,87].

The knockdown of Grx5 in HeLa cells resulted in mitochondrial iron overload and reduced cytosolic iron levels, and knockdown of Grx5 in erythrocytes resulted in the decreased expression of ferritin, delta-aminolevulinate synthase 2, and ferrochelatase [30]. In addition, Grx5 can also form clusters with the BolA-like protein family in the cytoplasm and plays a role in the maturation of FeS proteins [91]. [2Fe–2S] in BolA1-Grx5 may be part of the electron transfer process, but is not suitable for FeS transport [92]. [2Fe–2S] BolA3-Grx5 heterocomplexes may be more prone to FeS cluster transport [91]. Mutations in BolA3 cause hyperglycemic metabolic acidosis and deficiency of respiratory complexes and lipoic acid-conjugating enzymes [93]. To date, multiple cases of two distinct phenotypes, including sideroblastic anemia and variant non-ketotic hyperglycinemia, caused by mutations in the Grx5 gene have been reported [[94], [95], [96], [97], [98], [99], [100]]. Furthermore, an impaired FeS cluster synthesis causes increased intracellular iron and ROS, including superoxide (O_2_^•-^), H_2_O_2_, and the hydroxyl radical (HO•) via the iron starvation response and the Fenton reaction, potentially initiating ferroptosis (as reviewed in Refs. [88,101]). This oxidative, non-apoptotic cell decay is caused by lipid peroxidation and subsequent plasma membrane rupture [102]. Consequently, the inhibition of Grx5 renders cancer cells resistant to chemotherapeutic agents more susceptible to ferroptosis [103].

In summary, Grxs are essential for a broad range of cellular pathways and functions. Little is known about their significance for the pancreatic beta-cell and in diabetes mellitus with its complications. Therefore, the purpose of this review is to give an overview of the available literature.

## 2 Literature search

**Fig. 2:**
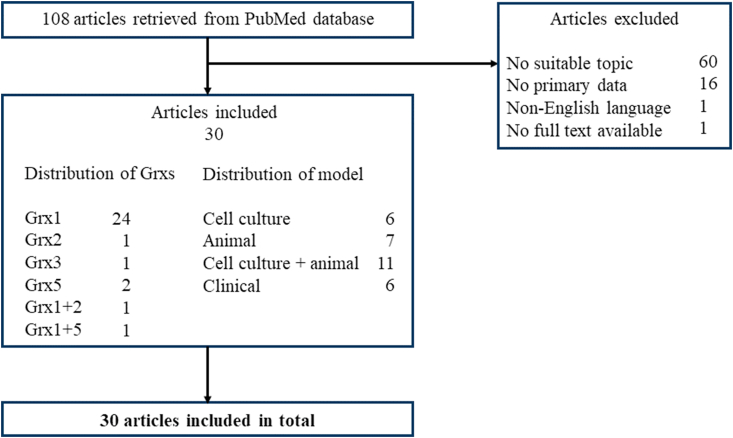
Overview of the literature search on PubMed.

The search of literature was conducted on PubMed and was last revised on the 3^rd^ of December 2023. The search term “(glutaredoxin* OR grx OR glrx OR thioltransferase) AND (diabetes OR islet* OR “beta cell*” OR pancreatic OR pancreas OR neuropathy OR nephropathy OR retinopathy OR “kidney disease”)” yielded a total of 108 primary articles. The publication dates ranged from February 1985 to October 2023. After screening the available literature, 30 publications were relevant to this review, among which 6 were about Grxs and beta-cells, 12 about Grxs and diabetic cardiovascular complications, 4 about Grxs and diabetic retinopathy or cataract, 2 about Grxs and non-alcoholic fatty liver disease, 3 about Grxs and diabetic nephropathy, and 2 about Grxs and diabetic central nervous system complications. Exclusion criteria were: no suitable topic (60 exclusions), non-English writing (1 exclusion), reviews with no primary data (16 exclusions), and no full text available (1 exclusion). Fig. 2 shows the flow chart of the literature search. A list of all screened articles is provided as supplementary material.

## 3 Glutaredoxins in diabetes mellitus and its complications

### 3.1 Glutaredoxins and pancreatic beta-cells

Pancreatic islets are among the tissues with the highest metabolic activity [104,105]. The synthesis, storage, and release of insulin by pancreatic beta-cells is highly dependent on mitochondrial oxidative metabolism [2]. Especially under diabetic conditions, hyperglycemia, free fatty acids, and inflammatory cytokines all contribute to an increase in ROS production at high metabolic activity levels through multiple pathways [106,107]. The expression level of classical antioxidant enzymes, such as superoxide dismutase, glutathione peroxidase, and catalase is low in the pancreas when compared with other tissues [106,108]. However, the mammalian Grx1, 2, 3, 5 and GR are differentially expressed in the Islets of Langerhans [23,[109], [110], [111], [112], [113]]. Godoy et al. were the first authors to systematically screen mouse tissues for redoxin expression. Accordingly, they described a strong expression of especially Grx1, 2 and 5 in the murine pancreas in their redox atlas of the mouse. Grx3 exhibited a nuclear localization pattern. Note, that also gamma-glutamylcysteine synthetase, the rate limiting enzyme in GSH synthesis, is highly expressed in the pancreatic beta-cells [114]. More detailed data is barely available. For Grx1, a higher mRNA expression was found in pancreatic beta-cells when compared with islet non-beta-cells and brain, lung, liver, adipose tissue, kidney, and skeletal muscle in rats. Grx1 protein is highly expressed in the islets, INS1-cells (rat insulinoma cells) and brain [115]. In pancreatic beta-cells, Grx1 is primarily found in the cytosol near the cell membrane [116].

Grxs are crucial for the maintenance of the cellular redox state but are also essential actors in many cellular functions beyond oxidative eustress. Previous studies from our group found a distinct protein and mRNA expression of Grxs in the islets of diabetic obese homozygous db-mice and non-diabetic lean control animals [113]. The most pronounced difference was detected for Grx1 and 5 [110,111]. Diabetic animals presented a loss of islet Grx1 and 5 when compared with controls, and there is also evidence that islet Grx5 and insulin, which are reduced by high fat diet (HFD), can be restored when switching to control diet. This mitigated Grx expression correlated with the diabetic phenotype of the animals and an increased production of H_2_O_2_ in their islets. Grx1 protein and mRNA expression in db/db-mouse islets were positively connected with islet count but negatively correlated with islet size and proliferation rate [111]. It is thus tempting to speculate that Grx1 has anti-apoptotic and pro-proliferative effects in pancreatic islets. A potential impact on glucose metabolism was indeed detected in Grx1 knockout mice [117,118]. Shao et al. found that compared with wild type (WT) mice, Grx1^−/−^-mice developed glucose intolerance and insulin resistance at 8 months of age when fed a standard diet [118]. Moreover, Wohua and Weiming reported that Grx2 knockout mice also showed significant insulin resistance, not only with elevated fasting blood glucose and fast insulin but also with considerably increased measurements in oral glucose tolerance (OGTT) and insulin tolerance testing (ITT) compared to WT mice when fed with HFD [53].

It is known that a high NADPH/NADP^+^ ratio caused by the pentose phosphate route [119], the cytosolic or mitochondrial citrate-pyruvate cycle [120,121], and the NAD^+^ kinase pathway [122] as well as experimental supplementation [115] enhances Ca^2+^ and ATP-mediated insulin exocytosis in beta-cells [115]. Remarkably, this can be enhanced by exogenous supplementation or endogenous overexpression of Grx1 as shown in rat islet beta-cells and INS-1832/13-cells and inhibited by Grx1 silencing [115,116]. Earlier studies found that parallel to the increased NADPH/NADP^+^ ratio, glucose enhanced the GSH/GSSG-ratio by increasing GSH and decreasing GSSG levels in a dose-dependent manner in isolated rat pancreas [123], and exogenous GSH increases glucose-induced insulin secretion in isolated rat pancreas in a dose-dependent manner [124]. In recent years, clinical trials have found that oral GSH supplementation can benefit the function of beta-cells in diabetic patients, enhance insulin secretion, and reduce HbA1c [125,126]. Since Grx1 requires GSH as a cofactor, a high GSH environment may increase Grx1 activity.

The available data imply that Grx1 is an important factor for insulin secretion. Since Grxs were downregulated in murine models of diabetes and cell culture exposed to fatty acids, it can be speculated about a link between Grx-deficiency and the impaired secretory machinery of the beta-cell. Grxs are involved in complex cellular networks and pathways. It is thus unclear whether the loss of Grxs simply reflects cellular stress and/or damage in general, and whether treatment approaches aiming to maintain Grx concentration and/or activity have impact on the complex cellular redox system and eventually on the endocrine function of the beta-cell. The literature research did not reveal *in vivo* treatment studies employing a reconstitution of Grxs in diabetes. Data are summarized in Table 1.

**Table 1:** Overview on Grxs and beta-cell/islet function in diabetes.

[23] 2011 Godoy et al.	Mouse pancreas	Grx1, 2, 3 and 5, and gGCS are expressed in pancreatic tissue; Grx1, 2, 5, and gGCS: strongest expression Grx3 and 5: nuclear staining pattern
[115] 2005 Ivarsson et al.	Rat islets and INS1-cells	Grx1 is highly expressed
[111] 2017 Petry et al.	db/db islets	Grx1, 2, 3, 5 ↓ ROS ↑
[110] 2018 Petry et al.	MIN6-cells + hypoxia	Grx5 ↓
[112] 2022 Petry et al.	C57BL/6 + HFD islets	Grx5 ↓
	MIN6 + oleic/palmitic acid	Grx5 ↓
[118] 2017 Shao et al.	C57BL/6NJ Grx1^−/−^ mice	glucose intolerance↑ insulin resistance ↑
[53] 2019 Wohua and Weiming	C57BL/6 Grx2^−/−^ mice + HFD	blood glucose ↑ insulin resistance ↑
[115] 2005 Ivarsson et al.	Rat beta-cells, Grx1	NADPH-dependent insulin secretion ↑
[116] 2009 Reinbothe et al.	INS-1832/13 cells/primary rat islets with Grx1-siRNA	GSIS ↓
	INS-1832/13 + Grx1-OE	GSIS ↑

gGCS: γ-glutamyl cysteinyl synthase; HFD: high fat diet; OE: overexpression; ↑: increase; ↓: decrease.

#### 3.1.1 Glutaredoxin-regulated pathways and mechanisms in the beta-cell

Grx1 regulates several critical proteins and processes involved in cell survival and death via deglutathionylation, such as Fas, caspase-3, PKC-alpha, inhibitor of nuclear factor kappa-B kinase subunit beta (IKKβ), NF-κβ, Adenosine monophosphate-activated protein kinase (AMPK), PTP1B, and aldose reductase (as reviewed in Ref. [127]). These thiol switches have been identified, however, not fully characterized in the context of beta-cell pathology. The available data on the current knowledge will be summarized. Please refer to other review articles that address their general significance for diabetes, including the pancreas and other metabolic organs [[128], [129], [130], [131], [132]].

##### 3.1.1.1 PTP1B

PTP1B inhibits the insulin signal transduction through dephosphorylation of insulin receptor [133] and insulin receptor substrate [134]. Silencing of PTP1B increases the proliferation rate and glucose-stimulated insulin secretion of beta-cells [135].

Interestingly, the active site Cys215 can undergo different types of oxidative modifications that can inhibit its catalytic activity reversibly or irreversibly [136]. A mutation of its active site Cys residue renders PTP1B catalytically inactive [137]. Recombinant PTP1B inactivated by diamide/GSH or GSSG can be reactivated by Grx1 [45], indicating that inhibiting Grx1 can suppress the activation of PTP1B and indirectly inhibit insulin transduction. Further research is required to elucidate the interaction of PTP1B and Grx1 in beta-cells *in vivo*. As Agrawal et al. summarized in a current review, PTP1B inhibitors have the potential to be used as antidiabetic agents [138]. Oxidative modifications of the enzyme might possibly interfere with the binding of PTPB1 inhibitors.

##### 3.1.1.2 PKC

PKC-alpha promotes insulin secretion by maintaining calcium channels [139]. The activation of the PKC pathway can inhibit apoptosis of beta-cells [140]. PKC is inactivated upon glutathionylation and Grx1 has been shown to restore its function [141]. It is tempting to speculate about a possible link between Grx1 and the preservation of beta-cell function, which has not yet been studied.

##### 3.1.1.3 NF-κB

The NF-κB signaling pathway includes the canonical pathway mediating inflammatory responses as well as non-canonical pathways involved in immune cell differentiation and maturation and secondary lymphoid organogenesis [142]. In pancreatic beta-cells, both signaling pathways can be activated and interact with each other [143]. Anti-apoptotic and pro-apoptotic functions of NF-κB in beta-cells have been described, partly depending on the species. The non-canonical NF-κB pathway has been shown to be pro-apoptotic and pro-inflammatory in pancreatic beta-cells when activated by cytokines [144]. It has been reported that the IKKβ/NF-κB pathway is involved in non-esterified fatty acids (NEFA)-induced beta-cell dysfunction [145]. Inhibition of IKKβ activity in *P. obesus* protects against diet-induced diabetes and decreases IL-1β induced ROS detected by employing the 2′,7′-Dichlorodihydrofluorescein diacetate (DCFH-DA) method (mainly HO•, H_2_O_2_, and the peroxynitrite anion [ONOO^−^]/radical [ROO•] [146]), loss of insulin production, and beta-cell death *in vitro* [147]. Beta-cell conditional-specific blockade of NF-κB can protect beta-cells from streptozotocin (STZ)-induced diabetes [148].

However, there is also evidence for a protective effect of NF-κB on beta-cells. Studies suggest that its activation can inhibit cytokine-induced insulin secretion defects and beta-cell death [[149], [150], [151]], induce the activation of the major anti-apoptotic gene A20 in beta-cells [152], and that overexpression of the NF-κB subunit c-Rel in human islets prevents caspase 3 activation and cell death triggered by cytokines and STZ [153]. Inhibition of NF-κB expression in beta-cells accelerated the development of autoimmune diabetes in non-obese diabetic (NOD) mice [154]. Our group found that cytokine-induced NF-κB activation had no effect on islet-cell viability under normoxic conditions but had a significant pro-apoptotic effect under hypoxic conditions [155]. Beta-cells become hypoxic under diabetic hypermetabolic conditions [156], which increases apoptosis by cytokine-induced NF-κB activation, suggesting that the function of NF-κB in beta-cells depends on its activator and cellular environment. We have reported a decreased protein level of Grx5 in MIN6-cells exposed to hypoxia [110], and Grx1 is positively regulated by NF-κB in macrophages and lung epithelial cells [157]. However, the regulation of NF-κB by Grx1 in pancreatic beta-cells is poorly understood.

##### 3.1.1.4 AMPK

AMPK plays an important role in the pathogenesis and treatment of diabetes [158]. The main functions of AMPK include promoting the uptake of glucose and fatty acids in peripheral tissues, inhibiting the synthesis of fatty acids and glycogen, promoting glucose metabolism, and improving insulin resistance [159,160]. In beta-cells, AMPK is essential for mitochondrial synthesis and cell maturation [161,162]. Although some available data suggest that AMPK mitigates insulin secretion from human and murine islets and MIN6-cells [162,163], it is generally acknowledged that the activation of AMPK can reduce inflammation and counteract oxidative distress, promote the survival of beta-cells, improve insulin sensitivity and glucose homeostasis thereby ameliorating glucose homeostasis [164].

Dong et al. found that both oxidative distress and inhibition of ROS can activate AMPK [165]. In rats, which were rendered diabetic by a combination of HFD and STZ, phosphorylated AMPK was increased. The antioxidant agents apocynin and allopurinol counteracted the detrimental impact of cytosolic H_2_O_2_ while activating Grx1 and 2 and AMPK. Additionally, the upregulation of both Grxs was accompanied by an increased phosphorylation of AMPK. Silencing of Grx1 and 2 resulted in the loss of phosphorylated AMPK.

Further experiments indicate a reciprocal and dose-dependent relationship between H_2_O_2_ and Grx1 and 2. 10 μM of H_2_O_2_ activated AMPK in an AMP-dependent manner, but 1 mM impaired both AMPK and Grx1 and 2 activity.

In addition, Dong et al. also found that Grx1 and 2 silencing decreased intracellular phosphofructokinase (PFK)-1, pyruvate kinase, and the phospho-PFK-2/PFK-2 ratio, indicating that Grx1 and 2 might in this way take effect on glucose metabolism.

##### 3.1.1.5 FeS clusters

FeS clusters are essential to fundamental biological reactions [93]. In pancreatic beta-cells, FeS clusters are involved in the translation of proinsulin [166,167] and contribute to the synthesis of ATP as part of the respiratory complexes, which play an important role in the metabolic coupling mechanism of glucose-stimulated insulin secretion [168]. Both FeS proteins Grx3 and Grx5 are involved in the biosynthesis and assembly of FeS clusters [83,86,88,169,170]. Our group found a decreased *Grx5* mRNA expression in islets of ageing diabetic and obese homozygous db- and control mice with the decline in diabetic mice being more pronounced [111]. Godoy et al. had previously described a nuclear location pattern in their redox atlas of the mouse [23] which was persistent in lean mice but markedly less distinct in diabetic db-animals [110]. In theory, this might indicate a shift of Grx5 into the mitochondria.

Subsequent research indicated that a HFD could induce islet Grx5 depletion, a significant increase in ROS generation measured by DCFH-DA, and a mitigated insulin secretion in islets of C57Bl/6 mice, along with a diabetic and obese phenotype and increased circulating FFAs. Remarkably, the islet Grx5 content and the described metabolic deviations could be restored by a carbohydrate-rich rescue diet [112].

In vitro, the exposure of MIN6-cells to FFAs resulted in a decrease in Grx5 accompanied by a decreased insulin secretion, which was consistent with altered mitochondrial markers, i.e. ATP levels and the O_2_-flux through the respiratory chain [112]. This study suggests that mitochondrial dysfunction induced by lipotoxicity may be the potential mechanism between the loss of Grx5 in islets and the decrease of insulin secretion.

##### 3.1.1.6 Iron homeostasis and ferroptosis

Bruni et al. have shown that human pancreatic islets are susceptible to ferroptosis by the induction with Erastin, which resulted in a decline of viability as assessed by lactate dehydrogenase release. Inhibition by Ferrostatin-1 rescued islet function [171]. There are case reports linking human Grx5-deficiency with diabetes mellitus through an impaired iron metabolism with cellular iron overload [94,97]. Taken together, the presence of ferroptosis provides a possible mechanism for beta-cell death. Data on Grxs and ferroptosis is limited. Lee et al. reported an increased susceptibility of cancer cells to ferroptosis through inhibition of Grx5 [103], and another study found a protection of ferroptosis concomitant with an upregulation of Grx1 and 2 by a small molecule in the lens [172], but there is no data on the beta-cell. Since the number of publications on ferroptosis in the beta-cell is increasing [[173], [174], [175], [176]], insights in the role of Grxs are expected.

### 3.2 Glutaredoxins and diabetic complications

The main cause of morbidity and mortality from diabetes is long-term damage to the vasculature, resulting in macro- or microangiopathies. Typical clinical manifestations are diabetic nephropathy, retinopathy, and neuropathy, but also myocardial infarctions, stroke, heart failure, and cognitive impairment [177].

Via the activation of the polyol, PKC, and hexosamine pathway, increased formation of advanced glycation end products and mitochondrial dysfunction, chronic hyperglycemia causes increased ROS production, which is an important mediator of these diabetic complications [178].

#### 3.2.1 Diabetic cardiomyopathy

Diabetes-related cardiovascular disease is the leading cause of death in people with diabetes, mainly including coronary artery disease and myocardial infarction caused by macrovascular lesions. Further, heart failure caused by diabetic cardiomyopathy (DCM) is a common complication [179]. The main pathological mechanism of DCM includes chronic hyperglycemia, hyperinsulinemia, and insulin resistance [180] inducing cardiac hypertrophy and myocardial fibrosis [181]. Grx1 was considerably increased in the left ventricular myocardium and plasma of diabetic rats induced by STZ and a HFD, but GR and protein thiol levels were decreased [46,182]. Qi et al. suggest that Grx1, which is highly expressed in cardiac fibroblasts under high glucose levels, might counteract the occurrence of DCM by inhibiting the expression of matrix metalloproteinase-2 and -9 and the activation of the NF-κB signaling pathway in cardiac rat fibroblasts [46]. This hypothesis is based on a decreased mRNA expression of both TNF-α as well as NFκB in Grx1-treated cells compared with high glucose-treatment. Further confirmatory research is required. The upregulation of Grx1 might be seen as an adaptive response to oxidative distress which can alleviate the challenge. As GR declines, the function of the Grx system may be impaired.

The Na^+^/K^+^ pump plays an important role in cell ion homeostasis, electrical signaling, and membrane transport function. Its activity is decreased in the ventricular myocardium and peripheral nerves of STZ-induced diabetic rats [183]. There is evidence indicating a correlation between reduced Na^+^/K^+^ pump activity and the development of persistent hyperglycemia, DCM, retinopathy, and neuropathy in this animal model as well [[184], [185], [186], [187]]. Further studies revealed that the Na^+^/K^+^ pump’s activity is regulated by glutathionylation of its β1-subunit [188]. Protein deglutathionylation is associated with increased binding of the Na^+^/K^+^ pump to Grx1, as demonstrated by co-immunoprecipitation of Grx1 with its β1 subunit [189]. Karimi Galougahi et al. found that hyperglycemia induced by continuous subcutaneous infusion of the insulin receptor antagonist S961 in male New Zealand white rabbits reduced this co-immunoprecipitation of Grx1 and β1 subunits in cardiomyocytes and increased the Na^+^/K^+^ pump β1 subunit glutathionylation, inhibiting the ion flux. The β3-adrenoceptor agonist CL316243 restored both the pump and the co-immunoprecipitation with Grx1 in a state of hyperglycemia [190]. However, as Black comments, the exact significance of Grx1, its relocation and the translational potential of the β-adrenoreceptor is still unclear [191].

Moreover, Coenzyme Q10 (CoQ10) was shown to prevent diabetic cardiac complications. In the left ventricle of STZ-induced diabetic mice, NADPH oxidase and markers for oxidative distress, such as O2•-, were upregulated, resulting in left ventricle diastolic dysfunction and cardiomyocyte hypertrophy. After 8 weeks of treatment with intraperitoneal injection or oral CoQ10 supplementation, NADPH oxidase-driven oxidative distress, inflammation, and apoptosis were attenuated, diastolic function was improved, and cardiac remodeling was limited in the left ventricle myocardium of diabetic mice compared with controls. It is suggested that CoQ10 supplementation can be used as an adjuvant treatment for DCM [192,193]. Grx1 was shown to catalyze the reduction of CoQ10 by GSH in a small cohort [160]. In patients suffering from type 2 diabetes, CoQ10 plasma levels were elevated and accompanied with lower Grx1 activity [194]. Oral administration of CoQ10 resulted in decreased extracellular Grx1, increased intracellular Grx1 protein and mRNA, and decreased serum Grx1 activity and total antioxidant capacity [195]. This is consistent with a higher plasma Grx1 activity in human patients with abnormal blood glucose levels or type 2 diabetes when compared with healthy subjects [196]. Plasma Grx1 activity may thus reflect the response to oxidative distress. Since there is no data indicating a general benefit from CoQ10 supplementation, the relevance of its impact on Grx1 does not appear to have clinical implications so far, at least for a duration of 12 weeks. Further, the authors report no difference between the studied subgroups (type 1 and type 2 diabetes, subjects treated with a statin).

#### 3.2.2 Coronary artery disease

Grx1 was found to be expressed in human coronary arteries and in macrophages infiltrating atherosclerotic lesions. In human coronary artery smooth muscle cells, H_2_O_2_ led to a significant increase in the expression of Grx1. In human coronary arteries, an enhanced Grx1 expression correlated with increased ROS [197].

Protein S-glutathionylation (PrS-SG) was found to be significantly increased in vascular EC isolated from type 2 diabetic patients compared with non-diabetic patients [198]. Exposure of human aortic EC to high concentrations of glucose and palmitic acid induced endothelial PrS-SG and EC dysfunction. Interestingly, the overexpression of Grx1 mitigated the PrS-SG and improved aortic endothelial barrier function in response to metabolic stress [198].

Further, decreased NO levels are critically entangled in diabetic vascular complications (as reviewed in Ref. [178]). Endothelial nitric oxide synthase (eNOS) is responsible for the NO production in vascular EC, and uncoupling of eNOS by PrS-SG adversely affects vascular function [199,200]. In bovine aortic EC, Grx1 was shown to reverse the PrS-SG of eNOS and restore its activity in the presence of GSH. The inhibition or gene silencing of Grx1 increased eNOS PrS-SG and decreased cellular NO production [201]. In coronary artery EC, treatment with Grx1 can protect cells from high glucose-induced protein carbonylation and apoptosis and reverse the high glucose-induced decrease in phospho-eNOS and NO levels [40].

Inconsistent with the increased glutathionylation of eNOS in hyperglycemic rabbit aorta, co-immunoprecipitation of eNOS/Grx1 and β1subunit/Grx1 was significantly diminished under hyperglycemic conditions [202], suggesting that hyperglycemia may impair Grx1 activity and increase eNOS glutathionylation. However, at the same concentration of Grx1, treatment of β3-Adrenoceptor agonist increased co-immunoprecipitation of eNOS/Grx1 and β1 subunit/Grx1 [202]. The β3-Adrenoceptor agonists may thus enhance Grx1-mediated deglutathionylation.

Grx1 is also involved in the cellular signaling of EC. High glucose activates the JNK/NF-κB signaling pathway and dephosphorylation of Akt, leading to apoptosis of human vascular EC [203,204]. Grx1 was shown to counteract this process [40]. Upregulation of Grx1 expression by Grx1 gene therapy reduced ischemia/reperfusion-mediated myocardial infarct area and cardiomyocyte death in diabetic hearts [44]. Further studies found that Grx1 gene therapy negatively regulates the ASK-1/JnK/p38 MAPK signaling pathway to inhibit apoptosis, but positively regulates the survival signaling via Akt-FoxO-1 and e-NOS and the expression of heme oxygenase-1 to switch the death signal into a survival signal [44]. In cardiac complications associated with ischemia-reperfusion in diabetic hearts, aldose reductase is activated in ischemia and subsequently inactivated during early reperfusion, which could be restored by Grx1 [205].

#### 3.2.3 Peripheral arterial disease

The prognosis for diabetic peripheral artery complications is poor, and post-ischemic angiogenesis deficiency may worsen the prognosis [206]. Angiogenesis is regulated by vascular endothelial growth factor (VEGF), which mediates angiogenesis via the activation of fms-like tyrosine kinase 1 (Flt-1) and fetal liver kinase 1 (Flk-1), with the latter inducing the main pro-angiogenic signal [207]. The soluble splice variant of Flt-1 (sFlt-1) binds VEGF with a higher affinity, thereby inhibiting Flk-1-mediated angiogenesis [208]. Following surgical induction of unilateral hindlimb ischemia in HFD-induced-diabetic mice, sFlt-1 was increased to a higher extent than Flt-1, thus limiting angiogenesis [209]. Grx1-overexpressing EC from transgenic mice showed an increased sFlt1 secretion in comparison with the controls. Following hindlimb ischemic surgery, these mice had attenuated revascularization and mitigated EC migration. Further data suggest that Grx1 overexpression in EC induces sFlt-1 expression by activating the Wnt5a-sFlt-1 pathway through NF-κB signaling [210]. Li et al. found that the plasmid-induced overexpression of VEGF can promote angiogenesis after hindlimb ischemia in mice which were rendered diabetic by HFD [206]. The knockdown of sFlt-1 by siRNA rescued the Grx1-induced attenuation of EC migration, reversed the suppressed network formation, and restored the reduced EC proliferation in human EC overexpressing Grx1 [210]. The authors propose the induction of sFlt by Grx1 through Wnt5a as a potential reason for impaired revascularization in limb ischemia. Thus, the clinical outcome of an increase in Grx1 would be detrimental in this context. Of note, the overexpression is artificial, not taking into account the physiological regulation of Grxs in ischemia. Therefore, conclusions should be drawn very carefully.

The reviewed data on the role of Grxs in cardiovascular complications of diabetes mellitus were obtained from several different models and are not consistent. As reviewed by Andreadou et al., there is more data on cardiovascular disease independent of diabetes mellitus [211]. The authors conclude that Grx1 has a protective role in cardiac hypertrophy, ischemia/reperfusion injury, and heart failure. However, there are contradictory data depending on the respective model, e.g., in limb revascularization Grx1 was detrimental to revascularization, suggesting that the significance of Grxs depends on the respective organ/tissue and the involved mechanisms. These comprise angiogenesis and protection from oxidative distress-induced apoptosis as well as PrS-SG [211]. As described by Han et al., a crucial function of Grx1 in the vasculature might be maintaining vascular barrier by mitigating PrS-SG [198]. Interestingly, another study found that in a model of mouse hindlimb ischemia, stabilization of hypoxia-inducible factor (HIF)-1α by oxidative modification was beneficial to revascularization. Accordingly, mitigating these protein modifications by Grx1 led to opposing results. Silencing of Grx1 improved revascularization [212]. Similar outcomes were found by Cohen et al. [213]. This indicates that research aiming to shed light on the translational potential of Grxs requires a thorough knowledge of the targeted tissue and physiological as well as pathological conditions. Otherwise, detrimental effects might occur. A summary of the reviewed data about the cardiovascular system is given in Table 2.

**Table 2:** Overview on Grx1 and cardiovascular disease in the context of diabetes.

[46] 2016 Qi et al.	Hyperglycemic human subjects, serum	Grx1 ↑
	Diabetic rats (HFD/STZ), serum	Grx1 ↑
	Diabetic rats, left ventricular myocardium	Grx1 ↑
	Rat cardiac fibroblasts + HG [25 mmol/l]	Grx1 ↑
	+ Grx1 + HG [50 mM]	TNF-α, NF-kB mRNA expression ↓
[190] 2015 Karimi Galoughahi et al.	Rabbit myocytes, induced hyperglycemia through insulin receptor antagonist S961	coimmunoprecipitation of Grx1 with the Na + -K+ pump β1-subunit ↓
	Treatment with the β3-Adrenoceptor agonist CL316243	coimmunoprecipitation ↑
[195] 2015 Montano et al.	Diabetic patients treated with Q10 for 12w serum	Grx1 activity ↓
	PBMC	Grx1 activity ↑
[196] 2014 Du et al.	Plasma of patients with diabetes mellitus typ 2 or abnormal glucose tolerance	Grx1 activity ↑ Grx1 ↑

[197] 2001 Okuda et al.	Human coronaries	Grx1 + ROS ↑
	Infiltrating macrophages	Grx1 ↑
	Human coronary artery smooth muscle cells + H_2_O_2_	Grx1 ↑
[198] 2016 Han et al.	Human aortic endothelial cells, Grx1 OE, palmitate [100 μM], HG [25 mM]	PrS-SG ↓ aortic endothelial barrier function ↓
[200] 2013 Chen et al.	Bovine aortic EC exogenous Grx1	PrS-SG of eNOS ↓ eNOS activity ↑
	inhibition/gene silencing of Grx1	PrS-SG of eNOS ↑ cellular NO production ↓
[40] 2014 Li et al.	Porcine coronary artery EC HG [25 mmol/l] + Grx1	protein carbonylation ↓ apoptosis ↓ phospho-eNOS ↑ NO levels ↑
[205] 2010 Wetzelberger et al.	Murine heart, reperfusion after ischemic injury + Grx1	aldose reductase activity ↑
[210] 2014 Murdoch et al.	Cardiac microvascular endothelial cells from Grx1^+/+^ mice	EC migration ↓ VEGF-induced network formation of EC ↓ sFlt1 ↑
	hindlimb ischemic surgery	revascularization ↓ EC migration ↓

HFD: high fat diet; STZ: streptozotocin; HG: high glucose; Q10: Coenzyme Q10; PBMC: peripheral blood mononuclear cell; OE: overexpression; EC: endothelial cells; ↑: increase; ↓: decrease.

#### 3.2.4 Diabetic retinopathy and cataract

Diabetic retinopathy (DR) is a prevalent chronic complication of diabetes and has been identified as the fifth leading cause of moderate to severe visual impairment and blindness globally [214]. The main pathologic feature is microangiopathy. Based on the presence or absence of neovascularization, DR can be classified as non-proliferative and proliferative, i.e., early and end stage, with or without macular edema [215]. The mechanisms of DR are mostly studied in terms of inflammation [216], apoptosis [217], vascular dysfunction [218], and destruction of neurovascular units [219]. Grx1 expression and activity have been described in ocular tissues such as the iris, ciliary body, cornea, lens, and retina [220]. However, in different eye tissues and conditions, upregulation of Grx1 appears to play a dual role. On one hand, Grx1 protects human retinal pigment epithelium (RPE) cells from oxidative distress-induced apoptosis, possibly related to its ability to stimulate Akt phosphorylation by preventing its glutathionylation [43]. In contrast, its amount and activity were significantly upregulated in retinal homogenates of STZ-induced diabetic rats and high-glucose-treated rat retinal Müller cells [221]. This upregulation is accompanied by NF-κB activation and increased expression of intercellular adhesion molecule 1 (ICAM-1), resulting in a pro-inflammatory response [221]. Notably, knockdown of Grx1 by siRNA in cells under high glucose conditions prevented ICAM-1 induction [221]. Further studies suggested that Grx1 regulates NF-κB activation and subsequent expression of ICAM-1 and IL-6 in retinal Müller cells by S-glutathionylation of IKKβ [222].

Conflicting data indicate that phosphorylation of AKT is associated with the formation of fibrotic membranes under high glucose conditions in RPE cells and plays an important role in the pathological process of DR [223]. Sustained endothelial activation of Akt induces structurally and functionally abnormal blood vessel formation [224]. Jiang et al. found that the expression of NF-κB increased with the duration of the disease in the retinal tissue of STZ-induced diabetic rats. It was closely related to neovascularization in the end stage of DR [225]. Therefore, despite the current inconclusive impact of Grx1 on diabetic retinopathy, the regulatory effect of Grx1 on AKT and NF-κB in diabetic retinopathy offers ample room for further research approaches.

The incidence of cataract in diabetic patients is significantly increased [226]. Although the pathogenesis of diabetic cataract is still not fully understood, there is some available data on the impact of oxidative distress. Markedly reduced levels of GSH concomitant with an increase in ROS as measured by the DCFH-DA method were detected in the lenses of galactose-fed rats, who presented with the rapid development of cataract [227]. Chan et al. achieved a reduction in the incidence of cataract by treatment with vitamin E and an exacerbation of cataract development in Glutathione peroxidase 1-deficient mice [227,228]. Two groups have studied the lenses of Grx1-deficient mice. Zhang et al. found a slightly higher opacity after treatment of lenses of knockout mice with 30 mM glucose for 48 h *ex vivo*. After diabetes was induced by STZ at the age of four months, the same observation was made *in vivo*. Further, the knockout mice had an increased amount of PrS-SG when compared to the WT animals [229]. Löfgren et al. studied lens epithelial cells of Grx1-deficient mice ex vivo. They were characterized by an increased protein glutathionylation, diminished GSH pool, H_2_O_2_-induced apoptosis, decreased clearance of H_2_O_2_, and impaired proliferation. Remarkably, restoring of Grx1 via protein transfection protected the cells of H_2_O_2_-induced inactivation [230].

In summary, the available data on Grx1 and the diabetic eye disease is scarce and controversial. As reviewed by Ren and Léveillard [231], oxidative distress is seen as a pivotal factor in retinal disease, but the significance of the inflammatory response in the context of Grx1 is not yet clear. More basal research will need to precede translational studies. The data on Grx1 and diabetic eye disease can be found in Table 3.

**Table 3:** Overview on Grx1 and diabetic retinopathy and cataract.

[221] 2007 Shelton et al.	Retina of STZ-induced rats	Grx1 ↑ Grx1 activity ↑
	r-MC1 cells + high glucose (25 mM)	Grx1 ↑ ICAM-1 ↑ NF-κB activation ↑
	r-MC1 cells + Grx1 KO	ICAM expression ↓
[43] 2015 Liu et al.	ARPE-19 cells, Grx1 OE + H_2_O_2_	cytotoxicity ↓
[222] 2009 Shelton et al.	r-MC1 cells, Grx1 OE	NF-κB activation ↑ ICAM-1 ↑ IL-6 ↑ IKKβ S-glutathionylation ↑
[229] 2017 Zhang et al.	Lenses of Grx1^−/−^-mice + STZ/high glucose (30 mM) ex vivo	lens opacity ↑ PrS-SG ↑
[230] 2008 Löfgren et al.	Lens epithelial cells of Grx1^−/−^-mice	PrS-SG ↑ GSH pool ↓ H_2_O_2_-induced apoptosis ↓ clearance of H_2_O_2_ ↓ impaired proliferation ↓
	Grx1-rescue	H_2_O_2_-induced apoptosis ↓

KO: knockout; OE: overexpression; STZ: Streptozocin; ICAM-1: intercellular adhesion molecule 1; IKKβ: inhibitor of nuclear factor kappa-B kinase subunit beta; PrS-SG: Protein S-glutathionylation. ↑: increase; ↓: decrease.

#### 3.2.5 Diabetic nephropathy

Diabetic nephropathy is the most important cause of chronic kidney disease (CKD). The global prevalence is estimated at around 50% in patients with type 2 diabetes and 32% in type 1 [232,233]. Levin et al. studied Grx1 activity in 61 CKD patients of whom 16 suffered from diabetes (type not classified). The serum activity of Grx1 in the CKD patients was higher than in the control group. At the beginning of dialysis, diabetic patients had non-significantly higher Grx1 activity. Further analysis found no correlation between Grx1 and HbA1c. The authors speculate that this finding might be related to the reduced lifespan of red blood cells in CKD, resulting in a general decrease in HbA1c [234]. Another study included 114 insulin-dependent patients with diabetes and retinopathy or nephropathy and 72 healthy subjects. The platelet Grx1 activity was non-significantly lower in the patients when compared with the controls. However, it was significantly mitigated in these with microalbuminuria. There was no relation of Grx1 activity and retinopathy [235]. This is consistent with the findings of a reduced Grx1 activity in platelets of patients suffering from type 2 diabetes by Di Simplicio et al. [236]. Since there are no further data, the significance of these findings for CKD/diabetic nephropathy is unclear (Table 4).

**Table 4:** Overview on Grx1 and diabetic kidney disease.

[234] 2018 Levin et al.	Human serum, CKD	Grx1 activity ↑
	Human serum, CKD + DM	Grx1 activity -
[235] 2000 Seghieri et al.	Human platelets, diabetic kidney disease	Grx1 activity -
	Human platelets, microalbuminuria	Grx1 activity ↓

CKD: chronic kidney disease. ↑: increase; ↓: decrease; -: no significant change.

#### 3.2.6 Neurological complications

Diabetes affects neural tissue and cerebrovascular structures, leading to neurological dysfunction and various acute and chronic disorders [237]. A longitudinal cohort study with a follow-up time of 31.7 years showed that type 2 diabetes was associated with an increased incidence of dementia, and the younger the age of onset of diabetes, the higher the risk [238]. Wohua and Weiming found that HFD-fed Grx2 KO mice had a higher blood glucose and more pronounced insulin resistance. Interestingly, obvious learning and memory-related cognitive dysfunction occurred compared with HFD-fed WT mice [53]. Histological analysis revealed that the HFD resulted in a decrease in the number of surviving neurons, activation of glial cells, upregulation of cell inflammatory factors such as TNF-α, IL-6, and IL-1β in the hippocampus, an increase in H_2_O_2_ levels and mitochondrial ROS production detected by DCFH-DA, and a decrease in the GSK-3β phosphorylation (inactive state). These effects were more distinct in the Grx2 KO mice [53]. GSK-3β is involved in the regulation of mitochondrial dysfunction, and inactivation of GSK-3β promotes mitochondrial energy metabolism and improves mitochondria-dependent apoptosis [239]. Similar results were obtained from primary astrocytes and murine microglial cells with induced Grx2-deficiency. These pathologies could be rescued by blocking GSK-3β with SB216763 [53]. There are multiple reports proposing inhibition of GSK-3β as a therapeutic strategy in neurological diseases [240,241] due to its significance for neuronal energy metabolism. Qiu et al. described a protective role of Grx1 against oxygen-glucose deprivation/reoxygenation-induced apoptosis and oxidative stress through GSK-3β/Nrf2 [242]. In a rat model of myocardial infarction, antioxidant treatment resulted in decreased activity of Grx1 and an increase in *p*-GSK-3 β in comparison with untreated animals [243]. These findings led to the conclusion that Grx1 might be involved in GSK-3β signaling. According to the available data it is however unclear whether Grxs are specifically involved in this pathway or whether the changes in Grx expression and/or activity reflect redox distress/inflammation. Regarding the brain, HFD is known to induce neuronal inflammation and cognitive impairment in mice independent of a Grx knockout [244,245]. Accordingly, in the brains of the previously mentioned HFD-fed C57Bl/6 mice, which featured a marked loss of islet Grx5 [[110], [111], [112],^246^], the mRNA expression of complex IV of the respiratory chain, citrate synthase, and glutathione peroxidase 1 as potential markers for enhanced inflammation, mitochondrial dysfunction, and oxidative distress were significantly increased. However, brain Grxs were not studied [246]. A summary is given in Table 5.

**Table 5:** Overview on Grx2 and neurological complications of diabetes.

[53] 2019 Wohua and Weiming	C57BL/6 Grx2^−/−^ mice + HFD	cognitive dysfunction ↑ surviving neurons ↓ glial cell activation ↑ TNF-α, IL-6, and IL-1β mRNA in the hippocampus ↑ H_2_O_2_ and mitochondrial ROS ↑ GSK-3β phosphorylation ↓
	BV2-/AST-cells, Grx2 siRNA, H_2_O_2_	mitochondrial dysfunction ↑ TNF-α and IL-1β mRNA ↑ phosphorylated GSK-3β ↓

GSK-3β: Glycogen Synthase Kinase 3 Beta. ↑: increase; ↓: decrease.

#### 3.2.7 Nonalcoholic fatty liver disease

Nonalcoholic fatty liver disease (NAFLD) is considered an overlooked diabetic complication [[247], [248], [249], [250]]. There is a mutual relation between NAFLD and type 2 diabetes. About 70% of people with type 2 diabetes have NAFLD [251]. NAFLD also promotes further development of chronic macrovascular and microvascular complications of diabetes [252]. Grx1 plays an important role in regulating oxidative eustress and metabolism in the liver. Its expression was decreased in patients with NAFLD as determined by liver biopsies [118]. Grx1^−/−^-mice exhibited increased hepatic lipid accumulation and hyperlipidemia on normal diet compared with WT mice, which could be ameliorated by adenovirus-mediated Grx1 gene supplementation [118]. Remarkably, the progression of NAFLD to nonalcoholic steatohepatitis (NASH) was accelerated in Grx1^−/−^-mice when fed a HFD compared with WT mice. Furthermore, hepatic sirtuin-1 (Sirt1) activity was reduced. It was restored by Grx1 gene supplementation [118], indicating that Sirt1 may be a target of Grx1-mediated lipid metabolism. Ahmad et al. reported similar results in Grx1^−/−^-mice. When Grx1 was deleted by CRISPR, serum triacylglycerol, total cholesterol, low-density lipoprotein cholesterol, alanine aminotransferase, and aspartate aminotransferase, as well as serum and liver TNF-α, IL-1β, IL-6, leptin, and lipopolysaccharide were increased, resulting in increased liver mass, necrosis, and inflammation [253,254]. Compared with the HFD WT mice, Grx2 KO mice also had significantly elevated serum triacylglycerol, total cholesterol, low-density lipoprotein cholesterol, and NEFA, as well as aggravated hepatic steatosis and hepatocyte swelling [53]. Accordingly, both Grx1 and 2 appear to be crucial for hepatic energy metabolism. It is an ongoing discussion whether NAFLD/NASH precedes diabetes mellitus type 2 or whether it is a consequence of the disease [255]. The reviewed data indicates that hepatic Grx1/2 deficiency promotes steatosis of the liver and consecutively glucose intolerance/diabetes. The literature research did not reveal data implying a decrease in liver Grxs following diabetes induction by other means. Since hepatic steatosis is not necessarily apparent in diabetes, it remains unclear whether hepatic Grxs are altered in diabetes mellitus in general. Therefore, the translational significance for diabetes remains unclear (Table 6).

**Table 6:** Overview on Grxs and fatty liver disease in the context of diabetes.

Grx1		
[118] 2017 Shao et al.	Human liver (NAFLD)	Grx1 protein ↓
	C57BL/6NJ Grx1^−/−^ mice	hyperlipidemia, liver lipid accumulation ↑ liver Sirt1 activity ↓
	Reconstitution of Grx1	plasma cholesterol ↓ liver mass ↓ liver steatosis ↓ liver Sirt1 activity ↑
	C57BL/6NJ Grx1−/− mice + HFD	NAFLD → NASH ↑
[253,254] 2019/20 Ahmad et al.	C57BL/6J Grx1^−/−^ mice	glucose tolerance ↓
	C57BL/6J Grx1^−/−^ mice, serum	triacylglycerol ↑ total cholesterol ↑ LDL-C ↑ HDL-C ↓ ALT ↑ AST ↑ TNF-α, IL-1β, IL-6 ↑ leptin ↑ lipopolysaccharide ↑
	C57BL/6J Grx1^−/−^ mice, liver	TNF-α, IL-1β, IL-6 ↑ mass, necrosis, inflammation ↑
Grx2		
[53] 2019 Wohua and Weiming	C57BL/6 Grx2^−/−^ mice + HFD serum	triacylglycerol ↑ total cholesterol ↑ LDL-C ↑ NEFA ↑
	liver	steatosis ↑ hepatocyte swelling ↑

NAFLD: Nonalcoholic fatty liver disease; NASH: non-alcoholic steatohepatitis; Sirt1: hepatic sirtuin-1; LDL-C: low-density lipoprotein cholesterol; HDL-C: high-density lipoprotein cholesterol; ALT: alanine aminotransferase; AST: aspartate aminotransferase; NEFA: non-esterified fatty acids. ↑: increase; ↓: decrease.

## 4 Summary

The glutaredoxin system is an important part of the basic mammalian cellular maintenance and preservation machinery required for cell survival and function. The disturbance of the physiological compartmentalized redox-signaling is an acknowledged factor in chronic metabolic and inflammatory disease [256]. Accordingly, oxidative distress [106,129,[257], [258], [259]], protein carbonylation [260], lipid peroxidation [261], and thiol oxidation [262] as promoted by gluco- and lipotoxicity are essential actors in the molecular pathophysiology of diabetes mellitus. Different dysregulated signaling pathways have been linked to the absence of Grxs and mostly changes in glutathionylation. However, the identification and characterization of substrates is rare. Moreover, the monothiol Grx3 and 5 might link the glutaredoxin system to diabetes through the iron metabolism, mitochondrial dysfunction, and ferroptosis. Iron overload is known to disrupt glucose homeostasis [263,264] as also clinically demonstrated in several case reports of human Grx5-deficiency [[94], [95], [96], [97], [98], [99], [100]].

As highlighted in this review, Grxs have been studied in most tissues relevant for diabetes and its complications, but there is little data, which were obtained employing many different models, available in total. The majority of studies found variations of Grx expression and/or activity in diabetic animal and cell culture models. Some authors could even ameliorate diabetes-induced pathologies on the molecular level by overexpression of/treatment with Grxs. Interestingly, the reviewed data indicate a relevance for diabetes mellitus independent from the clinical classification of the disease (i.e., type 1 or 2) which is apparent in patients and animals or modelled in cell culture/animal experiments. The available data on Grx1 concentration and activity in serum/plasma is more consistent. An increase was found in human and rat blood, and Montano et al. found a decrease in Grx1 activity after treatment with coenzyme Q10. One study measured an increased Grx1 activity in peripheral blood mononuclear cells (PBMC), another found a decrease in platelets. No other Grxs were studied in blood in diabetic models (Table 7). The origin and function of extracellular Grxs is not yet clear. It might be a mere reflection of the chronic inflammation which is apparent in the diabetic metabolism and associated oxidative distress. We have previously described a paracrine regulation of beta-cells by secreted Trx1 [265]. It would be interesting to see if Grx1 is secreted in situations of metabolic stress in the same manner.

**Table 7:** Overview about the literature on Grx1 and serum/blood cells of human subjects suffering from diabetes and diabetic rats.

[46] 2016 Qi et al.	Hyperglycemic human subjects, serum	Grx1 ↑
	Diabetic rats, serum	Grx1 ↑
[195] 2015 Montano et al.	diabetic patients treated with Q10 for 12 weeks - serum	Grx1 activity ↓
	PBMC	Grx1 activity ↑
[196] 2014 Du et al.	Plasma of patients with diabetes mellitus typ 2 or abnormal glucose tolerance	Grx1 activity ↑ Grx1 ↑
[236] 1995 Di Simplicio al.	Human platelets of insulin-dependent patients	Grx1 activity ↓

Q10: Coenzyme Q10; PBMC: peripheral blood mononuclear cell; OE: overexpression; EC: endothelial cells; ↑: increase; ↓: decrease.

**Fig. 3:**
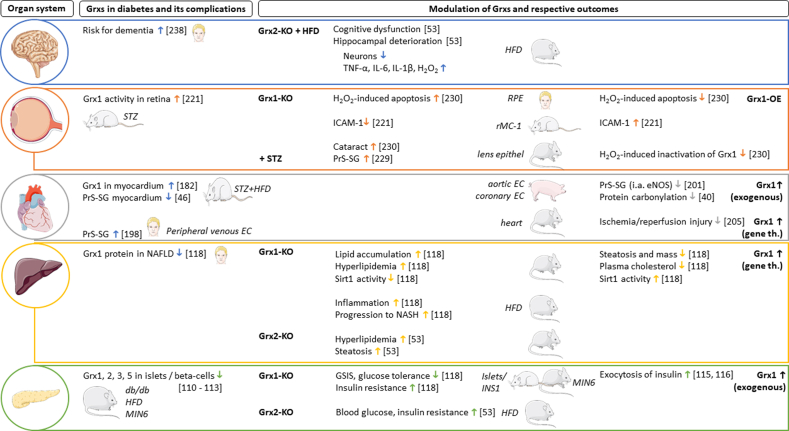
**Graphical summary of the reviewed data.** Grx: glutaredoxin; Grxs: glutaredoxins; KO: knockout; OE: overexpression; th.: therapy; HFD: high fat diet; STZ: Streptozocin; GSIS: glucose‐stimulated insulin secretion; PrS-SG: Protein S-glutathionylation; RPE: retinal pigment epithelium, rMC-1: retinal Muller Cell line-1; EC: endothelial cells; MIN6: mouse insulinoma 6; ICAM-1: intercellular adhesion molecule 1; eNOS: endothelial nitric oxide synthase; Sirt1: hepatic sirtuin-1; NAFLD: non-alcoholic fatty liver disease; NASH: non-alcoholic steatohepatitis. The figure was partly generated using Servier Medical Art, provided by Servier, licensed under a Creative Commons Attribution 3.0 unported license.

The main findings of this review are depicted in the graphical summary (Fig. 3).

In conclusion, the glutaredoxin system is a promising target for further basic research aiming to translate to therapeutic approaches with major questions unanswered so far. Judging by the reviewed data, precisely tailored approaches to address single Grx1 in target tissues would be required to achieve beneficial effects.

## Author contribution

M.Z.: conceptualization, methodology, original draft preparation, visualization.

E.M.H.: critical review, editing, visualization.

A.R.: critical review.

T.L.: conceptualization, critical review.

S.F.P.: conceptualization, editing, critical review, visualization, and supervision.

All authors have read and agreed to the published version of the manuscript.

The authors declare no conflict of interest. No funding was received for this review.

The graphical abstract and figure 3 were partly generated using Servier Medical Art, provided by Servier, licensed under a Creative Commons Attribution 3.0 unported license.

## Declaration of competing interest

The authors declare that they have no known competing financial interests or personal relationships that could have appeared to influence the work reported in this paper.

## Data Availability

No data was used for the research described in the article.

## References

[1] Sun H., Saeedi P., Karuranga S., Pinkepank M., Ogurtsova K., Duncan B.B., Stein C., Basit A., Chan J.C.N., Mbanya J.C., Pavkov M.E., Ramachandaran A., Wild S.H., James S., Herman W.H., Zhang P., Bommer C., Kuo S., Boyko E.J., Magliano D.J. (2022). IDF Diabetes Atlas: global, regional and country-level diabetes prevalence estimates for 2021 and projections for 2045. Diabetes Res. Clin. Pr..

[2] Rutter G.A., Pullen T.J., Hodson D.J., Martinez-Sanchez A. (2015). Pancreatic β-cell identity, glucose sensing and the control of insulin secretion. Biochem. J..

[3] Rossetti L., Giaccari A., DeFronzo R.A. (1990). Glucose toxicity. Diabetes Care.

[4] Römer A., Linn T., Petry S.F. (2021). Lipotoxic impairment of mitochondrial function in β-cells: a review. Antioxid. Basel Switz.

[5] Barlow J., Solomon T.P.J., Affourtit C. (2018). Pro-inflammatory cytokines attenuate glucose-stimulated insulin secretion from INS-1E insulinoma cells by restricting mitochondrial pyruvate oxidation capacity - novel mechanistic insight from real-time analysis of oxidative phosphorylation. PLoS One.

[6] Hanschmann E.-M., Godoy J.R., Berndt C., Hudemann C., Lillig C.H. (2013). Thioredoxins, glutaredoxins, and peroxiredoxins–molecular mechanisms and health significance: from cofactors to antioxidants to redox signaling. Antioxidants Redox Signal..

[7] Loreto Palacio P., Godoy J.R., Aktas O., Hanschmann E.-M. (2022). Changing perspectives from oxidative stress to redox signaling-extracellular redox control in translational medicine. Antioxid. Basel Switz..

[8] Laurent T.C., Moore E.C., Reichard P. (1964). Enzymatic synthesis of deoxyribonucleotides. IV. Isolation and characterization of thioredoxin, the hydrogen donor from escherichia coli B. J. Biol. Chem..

[9] Holmgren A. (1976). Hydrogen donor system for Escherichia coli ribonucleoside-diphosphate reductase dependent upon glutathione. Proc. Natl. Acad. Sci. U. S. A..

[10] Jones D.P. (2006). Redefining oxidative stress. Antioxidants Redox Signal..

[11] Sies H., Berndt C., Jones D.P. (2017). Oxidative stress. Annu. Rev. Biochem..

[12] Sies H. (2015). Oxidative stress: a concept in redox biology and medicine. Redox Biol..

[13] Luthman M., Eriksson S., Holmgren A., Thelander L. (1979). Glutathione-dependent hydrogen donor system for calf thymus ribonucleoside-diphosphate reductase. Proc. Natl. Acad. Sci. U. S. A.

[14] Martin J.L. (1995). Thioredoxin–a fold for all reasons. Structure.

[15] Bushweller J.H., Billeter M., Holmgren A., Wüthrich K. (1994). The nuclear magnetic resonance solution structure of the mixed disulfide between Escherichia coli glutaredoxin(C14S) and glutathione. J. Mol. Biol..

[16] Lillig C.H., Berndt C., Holmgren A. (2008). Glutaredoxin systems. Biochim. Biophys. Acta.

[17] Herrero E., de la Torre-Ruiz M.A. (2007). Monothiol glutaredoxins: a common domain for multiple functions. Cell. Mol. Life Sci. CMLS.

[18] Xing S., Lauri A., Zachgo S. (2006). Redox regulation and flower development: a novel function for glutaredoxins. Plant Biol. Stuttg. Ger.

[19] Alves R., Vilaprinyo E., Sorribas A., Herrero E. (2009). Evolution based on domain combinations: the case of glutaredoxins. BMC Evol. Biol..

[20] Couturier J., Jacquot J.-P., Rouhier N. (2009). Evolution and diversity of glutaredoxins in photosynthetic organisms. Cell. Mol. Life Sci. CMLS.

[21] Mondal S., Kumar V., Singh S.P. (2020). Phylogenetic distribution and structural analyses of cyanobacterial glutaredoxins (Grxs). Comput. Biol. Chem..

[22] Ogata F.T., Branco V., Vale F.F., Coppo L. (2021). Glutaredoxin: discovery, redox defense and much more. Redox Biol..

[23] Godoy J.R., Funke M., Ackermann W., Haunhorst P., Oesteritz S., Capani F., Elsässer H.-P., Lillig C.H. (2011). Redox atlas of the mouse. Immunohistochemical detection of glutaredoxin-, peroxiredoxin-, and thioredoxin-family proteins in various tissues of the laboratory mouse. Biochim. Biophys. Acta.

[24] Dammeyer P., Arnér E.S.J. (2011). Human Protein Atlas of redox systems - what can be learnt?. Biochim. Biophys. Acta.

[25] Johansson C., Lillig C.H., Holmgren A. (2004). Human mitochondrial glutaredoxin reduces S-glutathionylated proteins with high affinity accepting electrons from either glutathione or thioredoxin reductase. J. Biol. Chem..

[26] Begas P., Liedgens L., Moseler A., Meyer A.J., Deponte M. (2017). Glutaredoxin catalysis requires two distinct glutathione interaction sites. Nat. Commun..

[27] Trnka D., Engelke A.D., Gellert M., Moseler A., Hossain M.F., Lindenberg T.T., Pedroletti L., Odermatt B., de Souza J.V., Bronowska A.K., Dick T.P., Mühlenhoff U., Meyer A.J., Berndt C., Lillig C.H. (2020). Molecular basis for the distinct functions of redox-active and FeS-transferring glutaredoxins. Nat. Commun..

[28] Lillig C.H., Berndt C. (2013). Glutaredoxins in thiol/disulfide exchange. Antioxidants Redox Signal..

[29] Iwema T., Picciocchi A., Traore D.A.K., Ferrer J.-L., Chauvat F., Jacquamet L. (2009). Structural basis for delivery of the intact [Fe2S2] cluster by monothiol glutaredoxin. Biochemistry.

[30] Ye H., Jeong S.Y., Ghosh M.C., Kovtunovych G., Silvestri L., Ortillo D., Uchida N., Tisdale J., Camaschella C., Rouault T.A. (2010). Glutaredoxin 5 deficiency causes sideroblastic anemia by specifically impairing heme biosynthesis and depleting cytosolic iron in human erythroblasts. J. Clin. Invest..

[31] Shakamuri P., Zhang B., Johnson M.K. (2012). Monothiol glutaredoxins function in storing and transporting [Fe2S2] clusters assembled on IscU scaffold proteins. J. Am. Chem. Soc..

[32] Pai H.V., Starke D.W., Lesnefsky E.J., Hoppel C.L., Mieyal J.J. (2007). What is the functional significance of the unique location of glutaredoxin 1 (GRx1) in the intermembrane space of mitochondria?. Antioxidants Redox Signal..

[33] Lundberg M., Fernandes A.P., Kumar S., Holmgren A. (2004). Cellular and plasma levels of human glutaredoxin 1 and 2 detected by sensitive ELISA systems. Biochem. Biophys. Res. Commun..

[34] Kenchappa R.S., Ravindranath V. (2003). Glutaredoxin is essential for maintenance of brain mitochondrial complex I: studies with MPTP. Faseb. J..

[35] Song J.J., Rhee J.G., Suntharalingam M., Walsh S.A., Spitz D.R., Lee Y.J. (2002). Role of glutaredoxin in metabolic oxidative stress. Glutaredoxin as a sensor of oxidative stress mediated by H2O2. J. Biol. Chem..

[36] Hirota K., Matsui M., Murata M., Takashima Y., Cheng F.S., Itoh T., Fukuda K., Yo J., Junji Y. (2000). Nucleoredoxin, glutaredoxin, and thioredoxin differentially regulate NF-kappaB, AP-1, and CREB activation in HEK293 cells. Biochem. Biophys. Res. Commun..

[37] Daily D., Vlamis-Gardikas A., Offen D., Mittelman L., Melamed E., Holmgren A., Barzilai A. (2001). Glutaredoxin protects cerebellar granule neurons from dopamine-induced apoptosis by activating NF-kappa B via Ref-1. J. Biol. Chem..

[38] Reynaert N.L., van der Vliet A., Guala A.S., McGovern T., Hristova M., Pantano C., Heintz N.H., Heim J., Ho Y.-S., Matthews D.E., Wouters E.F.M., Janssen-Heininger Y.M.W. (2006). Dynamic redox control of NF-kappaB through glutaredoxin-regulated S-glutathionylation of inhibitory kappaB kinase beta. Proc. Natl. Acad. Sci. U. S. A..

[39] Yu H.T., Yue L.L., Zhang C.J. (2011). Grx1 antagonized high glucose-induced apoptosis in endothelial cells through inhibition of jnk pathway. Adv. Mater. Res..

[40] Li S., Sun Y., Qi X., Shi Y., Gao H., Wu Q., Liu X., Yu H., Zhang C. (2014). Protective effect and mechanism of glutaredoxin 1 on coronary arteries endothelial cells damage induced by high glucose. Bio Med. Mater. Eng..

[41] Mustafa Rizvi S.H., Shao D., Tsukahara Y., Pimentel D.R., Weisbrod R.M., Hamburg N.M., McComb M.E., Matsui R., Bachschmid M.M. (2021). Oxidized GAPDH transfers S-glutathionylation to a nuclear protein Sirtuin-1 leading to apoptosis. Free Radic. Biol. Med..

[42] Murata H., Ihara Y., Nakamura H., Yodoi J., Sumikawa K., Kondo T. (2003). Glutaredoxin exerts an antiapoptotic effect by regulating the redox state of Akt. J. Biol. Chem..

[43] Liu X., Jann J., Xavier C., Wu H. (2015). Glutaredoxin 1 (Grx1) protects human retinal pigment epithelial cells from oxidative damage by preventing AKT glutathionylation. Invest. Ophthalmol. Vis. Sci..

[44] Lekli I., Mukherjee S., Ray D., Gurusamy N., Kim Y.H., Tosaki A., Engelman R.M., Ho Y.S., Das D.K. (2010). Functional recovery of diabetic mouse hearts by glutaredoxin-1 gene therapy: role of Akt-FoxO-signaling network. Gene Ther..

[45] Barrett W.C., DeGnore J.P., König S., Fales H.M., Keng Y.F., Zhang Z.Y., Yim M.B., Chock P.B. (1999). Regulation of PTP1B via glutathionylation of the active site cysteine 215. Biochemistry.

[46] Qi X., Xu A., Gao Y., Shi Y., Sun X., Xu J., Liu J., Lan Q., Chang L., Zhang C., Yu H. (2016). Cardiac damage and dysfunction in diabetic cardiomyopathy are ameliorated by Grx1. Genet. Mol. Res..

[47] Gorelenkova Miller O., Behring J.B., Siedlak S.L., Jiang S., Matsui R., Bachschmid M.M., Zhu X., Mieyal J.J. (2016). Upregulation of glutaredoxin-1 activates microglia and promotes neurodegeneration: implications for Parkinson’s disease. Antioxidants Redox Signal..

[48] Cater M.A., Materia S., Xiao Z., Wolyniec K., Ackland S.M., Yap Y.W., Cheung N.S., La Fontaine S. (2014). Glutaredoxin1 protects neuronal cells from copper-induced toxicity, Biometals Int. J. Role Met. Ions Biol. Biochem. Med..

[49] Lönn M.E., Hudemann C., Berndt C., Cherkasov V., Capani F., Holmgren A., Lillig C.H. (2008). Expression pattern of human glutaredoxin 2 isoforms: identification and characterization of two testis/cancer cell-specific isoforms. Antioxidants Redox Signal..

[50] Hudemann C., Lönn M.E., Godoy J.R., Zahedi Avval F., Capani F., Holmgren A., Lillig C.H. (2009). Identification, expression pattern, and characterization of mouse glutaredoxin 2 isoforms. Antioxidants Redox Signal..

[51] Lundberg M., Johansson C., Chandra J., Enoksson M., Jacobsson G., Ljung J., Johansson M., Holmgren A. (2001). Cloning and expression of a novel human glutaredoxin (Grx2) with mitochondrial and nuclear isoforms. J. Biol. Chem..

[52] Gladyshev V.N., Liu A., Novoselov S.V., Krysan K., Sun Q.A., Kryukov V.M., Kryukov G.V., Lou M.F. (2001). Identification and characterization of a new mammalian glutaredoxin (thioltransferase), Grx2. J. Biol. Chem..

[53] Wohua Z., Weiming X. (2019). Glutaredoxin 2 (GRX2) deficiency exacerbates high fat diet (HFD)-induced insulin resistance, inflammation and mitochondrial dysfunction in brain injury: a mechanism involving GSK-3β. Biomed. Pharmacother..

[54] Enoksson M., Fernandes A.P., Prast S., Lillig C.H., Holmgren A., Orrenius S. (2005). Overexpression of glutaredoxin 2 attenuates apoptosis by preventing cytochrome c release. Biochem. Biophys. Res. Commun..

[55] Lillig C.H., Lönn M.E., Enoksson M., Fernandes A.P., Holmgren A. (2004). Short interfering RNA-mediated silencing of glutaredoxin 2 increases the sensitivity of HeLa cells toward doxorubicin and phenylarsine oxide. Proc. Natl. Acad. Sci. U. S. A..

[56] Kanaan G.N., Ichim B., Gharibeh L., Maharsy W., Patten D.A., Xuan J.Y., Reunov A., Marshall P., Veinot J., Menzies K., Nemer M., Harper M.-E. (2018). Glutaredoxin-2 controls cardiac mitochondrial dynamics and energetics in mice, and protects against human cardiac pathologies. Redox Biol..

[57] Wu H., Yu Y., David L., Ho Y.-S., Lou M.F. (2014). Glutaredoxin 2 (Grx2) gene deletion induces early onset of age-dependent cataracts in mice. J. Biol. Chem..

[58] Scalcon V., Folda A., Lupo M.G., Tonolo F., Pei N., Battisti I., Ferri N., Arrigoni G., Bindoli A., Holmgren A., Coppo L., Rigobello M.P. (2022). Mitochondrial depletion of glutaredoxin 2 induces metabolic dysfunction-associated fatty liver disease in mice. Redox Biol..

[59] Diotte N.M., Xiong Y., Gao J., Chua B.H.L., Ho Y.-S. (2009). Attenuation of doxorubicin-induced cardiac injury by mitochondrial glutaredoxin 2. Biochim. Biophys. Acta.

[60] Godoy J.R., Oesteritz S., Hanschmann E.-M., Ockenga W., Ackermann W., Lillig C.H. (2011). Segment-specific overexpression of redoxins after renal ischemia and reperfusion: protective roles of glutaredoxin 2, peroxiredoxin 3, and peroxiredoxin 6. Free Radic. Biol. Med..

[61] Karunakaran S., Saeed U., Ramakrishnan S., Koumar R.C., Ravindranath V. (2007). Constitutive expression and functional characterization of mitochondrial glutaredoxin (Grx2) in mouse and human brain. Brain Res..

[62] Schütte L.D., Baumeister S., Weis B., Hudemann C., Hanschmann E.-M., Lillig C.H. (2013). Identification of potential protein dithiol-disulfide substrates of mammalian Grx2. Biochim. Biophys. Acta.

[63] Taylor E.R., Hurrell F., Shannon R.J., Lin T.-K., Hirst J., Murphy M.P. (2003). Reversible glutathionylation of complex I increases mitochondrial superoxide formation. J. Biol. Chem..

[64] Beer S.M., Taylor E.R., Brown S.E., Dahm C.C., Costa N.J., Runswick M.J., Murphy M.P. (2004). Glutaredoxin 2 catalyzes the reversible oxidation and glutathionylation of mitochondrial membrane thiol proteins: implications for mitochondrial redox regulation and antioxidant DEFENSE. J. Biol. Chem..

[65] Daily D., Vlamis-Gardikas A., Offen D., Mittelman L., Melamed E., Holmgren A., Barzilai A. (2001). Glutaredoxin protects cerebellar granule neurons from dopamine-induced apoptosis by dual activation of the ras-phosphoinositide 3-kinase and jun n-terminal kinase pathways. J. Biol. Chem..

[66] Lillig C.H., Berndt C., Vergnolle O., Lönn M.E., Hudemann C., Bill E., Holmgren A. (2005). Characterization of human glutaredoxin 2 as iron-sulfur protein: a possible role as redox sensor. Proc. Natl. Acad. Sci. U. S. A..

[67] Berndt C., Hudemann C., Hanschmann E.-M., Axelsson R., Holmgren A., Lillig C.H. (2007). How does iron-sulfur cluster coordination regulate the activity of human glutaredoxin 2?. Antioxidants Redox Signal..

[68] Witte S., Villalba M., Bi K., Liu Y., Isakov N., Altman A. (2000). Inhibition of the c-Jun N-terminal kinase/AP-1 and NF-kappaB pathways by PICOT, a novel protein kinase C-interacting protein with a thioredoxin homology domain. J. Biol. Chem..

[69] Babichev Y., Isakov N., Mackiewicz A., Kurpisz M., Żeromski J. (2001). Adv. Exp. Med. Biol..

[70] Pham K., Pal R., Qu Y., Liu X., Yu H., Shiao S.L., Wang X., O’Brian Smith E., Cui X., Rodney G.G., Cheng N. (2015). Nuclear glutaredoxin 3 is critical for protection against oxidative stress-induced cell death. Free Radic. Biol. Med..

[71] Ohayon A., Babichev Y., Galperin M., Altman A., Isakov N. (2010). Widespread expression of PICOT in mouse and human tissues with predominant localization to epithelium. J. Histochem. Cytochem..

[72] Isakov N., Witte S., Altman A. (2000). PICOT-HD: a highly conserved protein domain that is often associated with thioredoxin and glutaredoxin modules. Trends Biochem. Sci..

[73] Babichev Y., Witte S., Altman A., Isakov N. (2001). Protein Modul. Cell. Signal..

[74] Jeong D., Cha H., Kim E., Kang M., Yang D.K., Kim J.M., Yoon P.O., Oh J.G., Bernecker O.Y., Sakata S., Le T.T., Cui L., Lee Y.-H., Kim D.H., Woo S.-H., Liao R., Hajjar R.J., Park W.J. (2006). PICOT inhibits cardiac hypertrophy and enhances ventricular function and cardiomyocyte contractility. Circ. Res..

[75] Jeong D., Kim J.M., Cha H., Oh J.G., Park J., Yun S.-H., Ju E.-S., Jeon E.-S., Hajjar R.J., Park W.J. (2008). PICOT attenuates cardiac hypertrophy by disrupting calcineurin-NFAT signaling. Circ. Res..

[76] Cha H., Kim J.M., Oh J.G., Jeong M.H., Park C.S., Park J., Jeong H.J., Park B.K., Lee Y.-H., Jeong D., Yang D.K., Bernecker O.Y., Kim D.H., Hajjar R.J., Park W.J. (2008). PICOT is a critical regulator of cardiac hypertrophy and cardiomyocyte contractility. J. Mol. Cell. Cardiol..

[77] Greene N.D.E., Leung K.-Y., Wait R., Begum S., Dunn M.J., Copp A.J. (2002). Differential protein expression at the stage of neural tube closure in the mouse embryo. J. Biol. Chem..

[78] Cheng N.-H., Zhang W., Chen W.-Q., Jin J., Cui X., Butte N.F., Chan L., Hirschi K.D. (2011). A mammalian monothiol glutaredoxin, Grx3, is critical for cell cycle progression during embryogenesis. FEBS J..

[79] Cha M.-K., Kim I.-H. (2009). Preferential overexpression of glutaredoxin3 in human colon and lung carcinoma. Cancer Epidemiol..

[80] Ohayon A., Babichev Y., Pasvolsky R., Dong G., Sztarkier I., Benharroch D., Altman A., Isakov N. (2010). Hodgkin’s lymphoma cells exhibit high expression levels of the PICOT protein. J. Immunot..

[81] Qu Y., Wang J., Ray P.S., Guo H., Huang J., Shin-Sim M., Bukoye B.A., Liu B., Lee A.V., Lin X., Huang P., Martens J.W., Giuliano A.E., Zhang N., Cheng N.-H., Cui X. (2011). Thioredoxin-like 2 regulates human cancer cell growth and metastasis via redox homeostasis and NF-κB signaling. J. Clin. Invest..

[82] Pandya P., Braiman A., Isakov N. (2019). PICOT (GLRX3) is a positive regulator of stress-induced DNA-damage response. Cell. Signal..

[83] Haunhorst P., Berndt C., Eitner S., Godoy J.R., Lillig C.H. (2010). Characterization of the human monothiol glutaredoxin 3 (PICOT) as iron-sulfur protein. Biochem. Biophys. Res. Commun..

[84] Haunhorst P., Hanschmann E.-M., Bräutigam L., Stehling O., Hoffmann B., Mühlenhoff U., Lill R., Berndt C., Lillig C.H. (2013). Crucial function of vertebrate glutaredoxin 3 (PICOT) in iron homeostasis and hemoglobin maturation. Mol. Biol. Cell.

[85] Lill R., Freibert S.-A. (2020). Mechanisms of mitochondrial iron-sulfur protein biogenesis. Annu. Rev. Biochem..

[86] Wingert R.A., Galloway J.L., Barut B., Foott H., Fraenkel P., Axe J.L., Weber G.J., Dooley K., Davidson A.J., Schmid B., Schmidt B., Paw B.H., Shaw G.C., Kingsley P., Palis J., Schubert H., Chen O., Kaplan J., Zon L.I. (2005). Deficiency of glutaredoxin 5 reveals Fe-S clusters are required for vertebrate haem synthesis. Nature.

[87] Banci L., Brancaccio D., Ciofi-Baffoni S., Del Conte R., Gadepalli R., Mikolajczyk M., Neri S., Piccioli M., Winkelmann J. (2014). [2Fe-2S] cluster transfer in iron-sulfur protein biogenesis. Proc. Natl. Acad. Sci. U.S.A..

[88] Ye H., Rouault T.A. (2010). Human iron-sulfur cluster assembly, cellular iron homeostasis, and disease. Biochemistry.

[89] Lill R., Hoffmann B., Molik S., Pierik A.J., Rietzschel N., Stehling O., Uzarska M.A., Webert H., Wilbrecht C., Mühlenhoff U. (2012). The role of mitochondria in cellular iron-sulfur protein biogenesis and iron metabolism. Biochim. Biophys. Acta.

[90] Mühlenhoff U., Braymer J.J., Christ S., Rietzschel N., Uzarska M.A., Weiler B.D., Lill R. (2020). Glutaredoxins and iron-sulfur protein biogenesis at the interface of redox biology and iron metabolism. Biol. Chem..

[91] Nasta V., Giachetti A., Ciofi-Baffoni S., Banci L. (2017). Structural insights into the molecular function of human [2Fe-2S] BOLA1-GRX5 and [2Fe-2S] BOLA3-GRX5 complexes. Biochim. Biophys. Acta Gen. Subj..

[92] Sen S., Rao B., Wachnowsky C., Cowan J.A. (2018). Cluster exchange reactivity of [2Fe-2S] cluster-bridged complexes of BOLA3 with monothiol glutaredoxins. Met. Integr. Biometal. Sci..

[93] Wachnowsky C., Fidai I., Cowan J.A. (2018). Iron-sulfur cluster biosynthesis and trafficking - impact on human disease conditions. Met. Integr. Biometal. Sci..

[94] Camaschella C., Campanella A., De Falco L., Boschetto L., Merlini R., Silvestri L., Levi S., Iolascon A. (2007). The human counterpart of zebrafish shiraz shows sideroblastic-like microcytic anemia and iron overload. Blood.

[95] Chiong M.A., Procopis P., Carpenter K., Wilcken B. (2007). Late-onset nonketotic hyperglycinemia with leukodystrophy and an unusual clinical course. Pediatr. Neurol..

[96] Baker P.R., Friederich M.W., Swanson M.A., Shaikh T., Bhattacharya K., Scharer G.H., Aicher J., Creadon-Swindell G., Geiger E., MacLean K.N., Lee W.-T., Deshpande C., Freckmann M.-L., Shih L.-Y., Wasserstein M., Rasmussen M.B., Lund A.M., Procopis P., Cameron J.M., Robinson B.H., Brown G.K., Brown R.M., Compton A.G., Dieckmann C.L., Collard R., Coughlin C.R., Spector E., Wempe M.F., Van Hove J.L.K. (2014). Variant non ketotic hyperglycinemia is caused by mutations in LIAS, BOLA3 and the novel gene GLRX5. Brain J. Neurol..

[97] Liu G., Guo S., Anderson G.J., Camaschella C., Han B., Nie G. (2014). Heterozygous missense mutations in the GLRX5 gene cause sideroblastic anemia in a Chinese patient. Blood.

[98] Daher R., Mansouri A., Martelli A., Bayart S., Manceau H., Callebaut I., Moulouel B., Gouya L., Puy H., Kannengiesser C., Karim Z. (2019). GLRX5 mutations impair heme biosynthetic enzymes ALA synthase 2 and ferrochelatase in Human congenital sideroblastic anemia. Mol. Genet. Metabol..

[99] Feng W.-X., Zhuo X.-W., Liu Z.-M., Li J.-W., Zhang W.-H., Wu Y., Han T.-L., Fang F. (2021). Case report: a variant non-ketotic hyperglycinemia with mutations: manifestation of deficiency of activities of the respiratory chain enzymes. Front. Genet..

[100] Sankaran B.P., Gupta S., Tchan M., Devanapalli B., Rahman Y., Procopis P., Bhattacharya K. (2021). GLRX5-associated [Fe-S] cluster biogenesis disorder: further characterisation of the neurological phenotype and long-term outcome. Orphanet J. Rare Dis..

[101] Dixon S.J., Stockwell B.R. (2014). The role of iron and reactive oxygen species in cell death. Nat. Chem. Biol..

[102] Dixon S.J., Lemberg K.M., Lamprecht M.R., Skouta R., Zaitsev E.M., Gleason C.E., Patel D.N., Bauer A.J., Cantley A.M., Yang W.S., Morrison B., Stockwell B.R. (2012). Ferroptosis: an iron-dependent form of nonapoptotic cell death. Cell..

[103] Lee J., You J.H., Shin D., Roh J.-L. (2020). Inhibition of glutaredoxin 5 predisposes cisplatin-resistant head and neck cancer cells to ferroptosis. Theranostics.

[104] Krzanowski J.J., Fertel R., Matschinsky F.M. (1971). Energy metabolism in pancreatic islets of rats. Studies with tolbutamide and hypoxia. Diabetes.

[105] Panten U., Zünkler B.J., Scheit S., Kirchhoff K., Lenzen S. (1986). Regulation of energy metabolism in pancreatic islets by glucose and tolbutamide. Diabetologia.

[106] Gehrmann W., Elsner M., Lenzen S. (2010). Role of metabolically generated reactive oxygen species for lipotoxicity in pancreatic β-cells. Diabetes Obes. Metabol..

[107] Andreyev A.Y., Kushnareva Y.E., Starkov A.A. (2005). Mitochondrial metabolism of reactive oxygen species. Biochem. Biokhimiia.

[108] Lenzen S., Drinkgern J., Tiedge M. (1996). Low antioxidant enzyme gene expression in pancreatic islets compared with various other mouse tissues. Free Radic. Biol. Med..

[109] Nagaoka Y., Iuchi Y., Ikeda Y., Fujii J. (2004). Glutathione reductase is expressed at high levels in pancreatic islet cells. Redox Rep. Commun. Free Radic. Res..

[110] Petry S.F., Sun L.M., Knapp A., Reinl S., Linn T. (2018). Distinct shift in beta-cell glutaredoxin 5 expression is mediated by hypoxia and lipotoxicity both *in vivo* and *in vitro*. Front. Endocrinol. Lausanne.

[111] Petry S.F., Sharifpanah F., Sauer H., Linn T. (2017). Differential expression of islet glutaredoxin 1 and 5 with high reactive oxygen species production in a mouse model of diabesity. PLoS One.

[112] Petry S.F., Römer A., Rawat D., Brunner L., Lerch N., Zhou M., Grewal R., Sharifpanah F., Sauer H., Eckert G.P., Linn T. (2022). Loss and recovery of glutaredoxin 5 is inducible by diet in a murine model of diabesity and mediated by free fatty acids *in vitro*. Antioxid. Basel Switz..

[113] Petry S.F. (2015).

[114] Tran P.O.T., Parker S.M., LeRoy E., Franklin C.C., Kavanagh T.J., Zhang T., Zhou H., Vliet P., Oseid E., Harmon J.S., Robertson R.P. (2004). Adenoviral overexpression of the glutamylcysteine ligase catalytic subunit protects pancreatic islets against oxidative stress. J. Biol. Chem..

[115] Ivarsson R., Quintens R., Dejonghe S., Tsukamoto K., in ’t Veld P., Renström E., Schuit F.C. (2005). Redox control of exocytosis: regulatory role of NADPH, thioredoxin, and glutaredoxin. Diabetes.

[116] Reinbothe T.M., Ivarsson R., Li D.Q., Niazi O., Jing X., Zhang E., Stenson L., Bryborn U., Renström E. (2009). Glutaredoxin-1 mediates NADPH-dependent stimulation of calcium-dependent insulin secretion. Mol. Endocrinol..

[117] Ho Y.-S., Xiong Y., Ho D.S., Gao J., Chua B.H.L., Pai H., Mieyal J.J. (2007). Targeted disruption of the glutaredoxin 1 gene does not sensitize adult mice to tissue injury induced by ischemia/reperfusion and hyperoxia. Free Radic. Biol. Med..

[118] Shao D., Han J., Hou X., Fry J., Behring J.B., Seta F., Long M.T., Roy H.K., Cohen R.A., Matsui R., Bachschmid M.M. (2017). Glutaredoxin-1 deficiency causes fatty liver and dyslipidemia by inhibiting sirtuin-1. Antioxidants Redox Signal..

[119] Giroix M.H., Sener A., Malaisse W.J. (1985). Pentose cycle pathway in normal and tumoral islet cells. FEBS Lett..

[120] Jensen M.V., Joseph J.W., Ronnebaum S.M., Burgess S.C., Sherry A.D., Newgard C.B. (2008). Metabolic cycling in control of glucose-stimulated insulin secretion. Am. J. Physiol. Endocrinol. Metab..

[121] Jitrapakdee S., Wutthisathapornchai A., Wallace J.C., MacDonald M.J. (2010). Regulation of insulin secretion: role of mitochondrial signalling. Diabetologia.

[122] Gray J.P., Alavian K.N., Jonas E.A., Heart E.A. (2012). NAD kinase regulates the size of the NADPH pool and insulin secretion in pancreatic β-cells. Am. J. Physiol. Endocrinol. Metab..

[123] Ammon H.P., Grimm A., Lutz S., Wagner-Teschner D., Händel M., Hagenloh I. (1980). Islet glutathione and insulin release. Diabetes.

[124] Ammon H.P., Klumpp S., Fuss A., Verspohl E.J., Jaeschke H., Wendel A., Müller P. (1989). A possible role of plasma glutathione in glucose-mediated insulin secretion: *in vitro* and *in vivo* studies in rats. Diabetologia.

[125] Bruggeman B.K., Storo K.E., Fair H.M., Wommack A.J., Carriker C.R., Smoliga J.M. (2019). The absorptive effects of orobuccal non-liposomal nano-sized glutathione on blood glutathione parameters in healthy individuals: a pilot study. PLoS One.

[126] Kalamkar S., Acharya J., Kolappurath Madathil A., Gajjar V., Divate U., Karandikar-Iyer S., Goel P., Ghaskadbi S. (2022). Randomized clinical trial of how long-term glutathione supplementation offers protection from oxidative damage and improves HbA1c in elderly type 2 diabetic patients. Antioxid. Basel Switz..

[127] Allen E.M., Mieyal J.J. (2012). Protein-thiol oxidation and cell death: regulatory role of glutaredoxins. Antioxidants Redox Signal..

[128] Peng M.-L., Fu Y., Wu C.-W., Zhang Y., Ren H., Zhou S.-S. (2022). Signaling pathways related to oxidative stress in diabetic cardiomyopathy. Front. Endocrinol..

[129] Singh A., Kukreti R., Saso L., Kukreti S. (2022). Mechanistic insight into oxidative stress-triggered signaling pathways and type 2 diabetes. Molecules.

[130] Geraldes P., King G.L. (2010). Activation of protein kinase C isoforms and its impact on diabetic complications. Circ. Res..

[131] Kang Q., Yang C. (2020). Oxidative stress and diabetic retinopathy: molecular mechanisms, pathogenetic role and therapeutic implications. Redox Biol..

[132] Berndt C., Lillig C.H., Holmgren A. (2007). Thiol-based mechanisms of the thioredoxin and glutaredoxin systems: implications for diseases in the cardiovascular system. Am. J. Physiol. Heart Circ. Physiol..

[133] Ravichandran L.V., Chen H., Li Y., Quon M.J. (2001). Phosphorylation of PTP1B at Ser(50) by Akt impairs its ability to dephosphorylate the insulin receptor. Mol. Endocrinol. Baltim. Md.

[134] Xue B., Kim Y.-B., Lee A., Toschi E., Bonner-Weir S., Kahn C.R., Neel B.G., Kahn B.B. (2007). Protein-tyrosine phosphatase 1B deficiency reduces insulin resistance and the diabetic phenotype in mice with polygenic insulin resistance. J. Biol. Chem..

[135] Fernandez-Ruiz R., Vieira E., Garcia-Roves P.M., Gomis R. (2014). Protein tyrosine phosphatase-1B modulates pancreatic β-cell mass. PLoS One.

[136] van Montfort R.L.M., Congreve M., Tisi D., Carr R., Jhoti H. (2003). Oxidation state of the active-site cysteine in protein tyrosine phosphatase 1B. Nature.

[137] Scapin G., Patel S., Patel V., Kennedy B., Asante-Appiah E. (2001). The structure of apo protein-tyrosine phosphatase 1B C215S mutant: more than just an S--> O change, Protein Sci. Publ. Protein Soc..

[138] Agrawal N., Dhakrey P., Pathak S. (2023). A comprehensive review on the research progress of PTP1B inhibitors as antidiabetics. Chem. Biol. Drug Des..

[139] Warwar N., Efendic S., Ostenson C.-G., Haber E.P., Cerasi E., Nesher R. (2006). Dynamics of glucose-induced localization of PKC isoenzymes in pancreatic beta-cells: diabetes-related changes in the GK rat. Diabetes.

[140] Zhang L., Wang Y., Wang J., Liu Y., Yin Y. (2015). Protein kinase C pathway mediates the protective effects of glucagon-like peptide-1 on the apoptosis of islet β-cells. Mol. Med. Rep..

[141] Ward N.E., Stewart J.R., Ioannides C.G., O’Brian C.A. (2000). Oxidant-induced S-glutathiolation inactivates protein kinase C-alpha (PKC-alpha): a potential mechanism of PKC isozyme regulation. Biochemistry.

[142] Shih V.F.-S., Tsui R., Caldwell A., Hoffmann A. (2011). A single NFκB system for both canonical and non-canonical signaling. Cell Res..

[143] Meyerovich K., Ortis F., Cardozo A.K. (2018). The non-canonical NF-κB pathway and its contribution to β-cell failure in diabetes. J. Mol. Endocrinol..

[144] Meyerovich K., Fukaya M., Terra L.F., Ortis F., Eizirik D.L., Cardozo A.K. (2016). The non-canonical NF-κB pathway is induced by cytokines in pancreatic beta cells and contributes to cell death and proinflammatory responses *in vitro*. Diabetologia.

[145] Ivovic A., Oprescu A.I., Koulajian K., Mori Y., Eversley J.A., Zhang L., Nino-Fong R., Lewis G.F., Donath M.Y., Karin M., Wheeler M.B., Ehses J., Volchuk A., Chan C.B., Giacca A. (2017). IKKβ inhibition prevents fat-induced beta cell dysfunction *in vitro* and *in vivo* in rodents. Diabetologia.

[146] Setsukinai K., Urano Y., Kakinuma K., Majima H.J., Nagano T. (2003). Development of novel fluorescence probes that can reliably detect reactive oxygen species and distinguish specific species. J. Biol. Chem..

[147] Friberg J., Tonnesen M.F., Heller S., Pociot F., Bödvarsdottir T.B., Karlsen A.E. (2010). Inhibition of the nuclear factor-κB pathway prevents beta cell failure and diet induced diabetes in Psammomys obesus. PLoS One.

[148] Eldor R., Yeffet A., Baum K., Doviner V., Amar D., Ben-Neriah Y., Christofori G., Peled A., Carel J.C., Boitard C., Klein T., Serup P., Eizirik D.L., Melloul D. (2006). Conditional and specific NF-kappaB blockade protects pancreatic beta cells from diabetogenic agents. Proc. Natl. Acad. Sci. U. S. A..

[149] Giannoukakis N., Rudert W.A., Trucco M., Robbins P.D. (2000). Protection of human islets from the effects of interleukin-1beta by adenoviral gene transfer of an Ikappa B repressor. J. Biol. Chem..

[150] Heimberg H., Heremans Y., Jobin C., Leemans R., Cardozo A.K., Darville M., Eizirik D.L. (2001). Inhibition of cytokine-induced NF-kappaB activation by adenovirus-mediated expression of a NF-kappaB super-repressor prevents beta-cell apoptosis. Diabetes.

[151] Bae U.-J., Jang H.-Y., Lim J.M., Hua L., Ryu J.-H., Park B.-H. (2015). Polyphenols isolated from Broussonetia kazinoki prevent cytokine-induced β-cell damage and the development of type 1 diabetes. Exp. Mol. Med..

[152] Liuwantara D., Elliot M., Smith M.W., Yam A.O., Walters S.N., Marino E., McShea A., Grey S.T. (2006). Nuclear factor-kappaB regulates beta-cell death: a critical role for A20 in beta-cell protection. Diabetes.

[153] Mokhtari D., Barbu A., Mehmeti I., Vercamer C., Welsh N. (2009). Overexpression of the nuclear factor-κB subunit c-Rel protects against human islet cell death *in vitro*. Am. J. Physiol. Endocrinol. Metab..

[154] Ramakrishnan P., Yui M.A., Tomalka J.A., Majumdar D., Parameswaran R., Baltimore D. (2016). Deficiency of nuclear factor-κB c-rel accelerates the development of autoimmune diabetes in NOD mice. Diabetes.

[155] Chen C., Moreno R., Samikannu B., Bretzel R.G., Schmitz M.L., Linn T. (2011). Improved intraportal islet transplantation outcome by systemic IKK-beta inhibition: NF-κB activity in pancreatic islets depends on oxygen availability. Am. J. Transplant. Off. J. Am. Soc. Transplant. Am. Soc. Transpl. Surg..

[156] Sato Y., Endo H., Okuyama H., Takeda T., Iwahashi H., Imagawa A., Yamagata K., Shimomura I., Inoue M. (2011). Cellular hypoxia of pancreatic beta-cells due to high levels of oxygen consumption for insulin secretion *in vitro*. J. Biol. Chem..

[157] Aesif S.W., Kuipers I., van der Velden J., Tully J.E., Guala A.S., Anathy V., Sheely J.I., Reynaert N.L., Wouters E.F.M., van der Vliet A., Janssen-Heininger Y.M.W. (2011). Activation of the glutaredoxin-1 gene by nuclear factor κB enhances signaling. Free Radic. Biol. Med..

[158] Zhang B.B., Zhou G., Li C. (2009). AMPK: an emerging drug target for diabetes and the metabolic syndrome. Cell Metabol..

[159] Steinberg G.R., Kemp B.E. (2009). AMPK in health and disease. Physiol. Rev..

[160] Steinberg G.R., Hardie D.G. (2023). New insights into activation and function of the AMPK. Nat. Rev. Mol. Cell Biol..

[161] Jaafar R., Tran S., Shah A.N., Sun G., Valdearcos M., Marchetti P., Masini M., Swisa A., Giacometti S., Bernal-Mizrachi E., Matveyenko A., Hebrok M., Dor Y., Rutter G.A., Koliwad S.K., Bhushan A. (2019). mTORC1 to AMPK switching underlies β-cell metabolic plasticity during maturation and diabetes. J. Clin. Invest..

[162] Leclerc I., Woltersdorf W.W., da Silva Xavier G., Rowe R.L., Cross S.E., Korbutt G.S., Rajotte R.V., Smith R., Rutter G.A. (2004). Metformin, but not leptin, regulates AMP-activated protein kinase in pancreatic islets: impact on glucose-stimulated insulin secretion. Am. J. Physiol. Endocrinol. Metab..

[163] Richards S.K., Parton L.E., Leclerc I., Rutter G.A., Smith R.M. (2005). Over-expression of AMP-activated protein kinase impairs pancreatic {beta}-cell function *in vivo*. J. Endocrinol..

[164] Misra P., Chakrabarti R. (2007). The role of AMP kinase in diabetes. Indian J. Med. Reserve.

[165] Dong K., Wu M., Liu X., Huang Y., Zhang D., Wang Y., Yan L.-J., Shi D. (2016). Glutaredoxins concomitant with optimal ROS activate AMPK through S-glutathionylation to improve glucose metabolism in type 2 diabetes. Free Radic. Biol. Med..

[166] Santos M.C.F.D., Anderson C.P., Neschen S., Zumbrennen-Bullough K.B., Romney S.J., Kahle-Stephan M., Rathkolb B., Gailus-Durner V., Fuchs H., Wolf E., Rozman J., de Angelis M.H., Cai W.M., Rajan M., Hu J., Dedon P.C., Leibold E.A. (2020). Irp2 regulates insulin production through iron-mediated Cdkal1-catalyzed tRNA modification. Nat. Commun..

[167] Torres A.G., Batlle E., Ribas de Pouplana L. (2014). Role of tRNA modifications in human diseases. Trends Mol. Med..

[168] Marku A., Galli A., Marciani P., Dule N., Perego C., Castagna M. (2021). Iron metabolism in pancreatic beta-cell function and dysfunction. Cells.

[169] Johansson C., Kavanagh K.L., Gileadi O., Oppermann U. (2007). Reversible sequestration of active site cysteines in a 2Fe-2S-bridged dimer provides a mechanism for glutaredoxin 2 regulation in human mitochondria. J. Biol. Chem..

[170] Berndt C., Christ L., Rouhier N., Mühlenhoff U. (2021). Glutaredoxins with iron-sulphur clusters in eukaryotes - structure, function and impact on disease. Biochim. Biophys. Acta Bioenerg..

[171] Bruni A., Pepper A.R., Pawlick R.L., Gala-Lopez B., Gamble A.F., Kin T., Seeberger K., Korbutt G.S., Bornstein S.R., Linkermann A., Shapiro A.M.J. (2018). Ferroptosis-inducing agents compromise *in vitro* human islet viability and function. Cell Death Dis..

[172] Zhang J., Yu Y., Mekhail M.A., Wu H., Green K.N. (2022). A macrocyclic molecule with multiple antioxidative activities protects the lens from oxidative damage. Front. Chem..

[173] Markelic M., Stancic A., Saksida T., Grigorov I., Micanovic D., Velickovic K., Martinovic V., Savic N., Gudelj A., Otasevic V. (2023). Defining the ferroptotic phenotype of beta cells in type 1 diabetes and its inhibition as a potential antidiabetic strategy. Front. Endocrinol..

[174] Sun Y., Guo L.-Q., Wang D.-G., Xing Y.-J., Bai Y.-P., Zhang T., Wang W., Zhou S.-M., Yao X.-M., Cheng J.-H., Chang W.-W., Lv K., Li C.-X., Kong X. (2023). Metformin alleviates glucolipotoxicity-induced pancreatic β cell ferroptosis through regulation of the GPX4/ACSL4 axis, Eur. J. Pharmacol..

[175] Du Q., Wu X., Ma K., Liu W., Liu P., Hayashi T., Mizuno K., Hattori S., Fujisaki H., Ikejima T. (2023). Silibinin alleviates ferroptosis of rat islet β cell INS-1 induced by the treatment with palmitic acid and high glucose through enhancing PINK1/parkin-mediated mitophagy. Arch. Biochem. Biophys..

[176] Novoselova E.G., Lunin S.M., Khrenov M.O., Glushkova O.V., Novoselova T.V., Parfenyuk S.B. (2023). The possible role of Β-cell senescence in the development of type 2 diabetes mellitus, cell. Physiol. Biochem. Int. J. Exp. Cell. Physiol. Biochem. Pharmacol..

[177] American Diabetes Association (2009). Diagnosis and classification of diabetes mellitus. Diabetes Care.

[178] Giacco F., Brownlee M. (2010). Oxidative stress and diabetic complications. Circ. Res..

[179] Maack C., Lehrke M., Backs J., Heinzel F.R., Hulot J.-S., Marx N., Paulus W.J., Rossignol P., Taegtmeyer H., Bauersachs J., Bayes-Genis A., Brutsaert D., Bugger H., Clarke K., Cosentino F., De Keulenaer G., Dei Cas A., González A., Huelsmann M., Iaccarino G., Lunde I.G., Lyon A.R., Pollesello P., Rena G., Riksen N.P., Rosano G., Staels B., van Laake L.W., Wanner C., Farmakis D., Filippatos G., Ruschitzka F., Seferovic P., de Boer R.A., Heymans S. (2018). Heart failure and diabetes: metabolic alterations and therapeutic interventions: a state-of-the-art review from the translational research committee of the heart failure association-European society of cardiology, eur. Hear. J..

[180] Murtaza G., Virk H.U.H., Khalid M., Lavie C.J., Ventura H., Mukherjee D., Ramu V., Bhogal S., Kumar G., Shanmugasundaram M., Paul T.K. (2019). Diabetic cardiomyopathy - a comprehensive updated review. Prog. Cardiovasc. Dis..

[181] Asbun J., Villarreal F.J. (2006). The pathogenesis of myocardial fibrosis in the setting of diabetic cardiomyopathy. J. Am. Coll. Cardiol..

[182] Li X., Xu Z., Li S., Rozanski G.J. (2005). Redox regulation of Ito remodeling in diabetic rat heart. Am. J. Physiol. Heart Circ. Physiol..

[183] Kjeldsen K., Braendgaard H., Sidenius P., Larsen J.S., Nørgaard A. (1987). Diabetes decreases Na+-K+ pump concentration in skeletal muscles, heart ventricular muscle, and peripheral nerves of rat. Diabetes.

[184] Bay J., Kohlhaas M., Maack C. (2013). Intracellular Na^+^ and cardiac metabolism. J. Mol. Cell. Cardiol..

[185] Djemli-Shipkolye A., Coste T., Raccah D., Vague P., Pieroni G., Gerbi A. (2001). Na,K-atpase alterations in diabetic rats: relationship with lipid metabolism and nerve physiological parameters. Cell. Mol. Biol. Noisy–Gd. Fr..

[186] Di Leo M.A.S., Santini S.A., Cercone S., Lepore D., Gentiloni Silveri N., Caputo S., Greco A.V., Giardina B., Franconi F., Ghirlanda G. (2002). Chronic taurine supplementation ameliorates oxidative stress and Na+ K+ ATPase impairment in the retina of diabetic rats. Amino Acids.

[187] Hansen P.S., Clarke R.J., Buhagiar K.A., Hamilton E., Garcia A., White C., Rasmussen H.H. (2007). Alloxan-induced diabetes reduces sarcolemmal Na+-K+ pump function in rabbit ventricular myocytes. Am. J. Physiol. Cell Physiol..

[188] Figtree G.A., Liu C.-C., Bibert S., Hamilton E.J., Garcia A., White C.N., Chia K.K.M., Cornelius F., Geering K., Rasmussen H.H. (2009). Reversible oxidative modification: a key mechanism of Na+-K+ pump regulation. Circ. Res..

[189] Bibert S., Liu C.-C., Figtree G.A., Garcia A., Hamilton E.J., Marassi F.M., Sweadner K.J., Cornelius F., Geering K., Rasmussen H.H. (2011). FXYD proteins reverse inhibition of the Na+-K+ pump mediated by glutathionylation of its beta1 subunit. J. Biol. Chem..

[190] Karimi Galougahi K., Liu C.C., Garcia A., Fry N.A., Hamilton E.J., Figtree G.A., Rasmussen H.H. (2015). β3-Adrenoceptor activation relieves oxidative inhibition of the cardiac Na+-K+ pump in hyperglycemia induced by insulin receptor blockade. Am. J. Physiol. Cell Physiol..

[191] Black S.M. (2015). β3-Adrenoceptor, glutathionylation, and diabetic cardiomyopathy. Focus on “β3-Adrenoceptor activation relieves oxidative inhibition of the cardiac Na+-K+ pump in hyperglycemia induced by insulin receptor blockade,”. Am. J. Physiol. Cell Physiol..

[192] Huynh K., Kiriazis H., Du X.-J., Love J.E., Gray S.P., Jandeleit-Dahm K.A., McMullen J.R., Ritchie R.H. (2013). Targeting the upregulation of reactive oxygen species subsequent to hyperglycemia prevents type 1 diabetic cardiomyopathy in mice. Free Radic. Biol. Med..

[193] De Blasio M.J., Huynh K., Qin C., Rosli S., Kiriazis H., Ayer A., Cemerlang N., Stocker R., Du X.-J., McMullen J.R., Ritchie R.H. (2015). Therapeutic targeting of oxidative stress with coenzyme Q10 counteracts exaggerated diabetic cardiomyopathy in a mouse model of diabetes with diminished PI3K(p110α) signaling. Free Radic. Biol. Med..

[194] Forsberg E., Xu C., Grünler J., Frostegård J., Tekle M., Brismar K., Kärvestedt L. (2015). Coenzyme Q10 and oxidative stress, the association with peripheral sensory neuropathy and cardiovascular disease in type 2 diabetes mellitus. J. Diabetes Complicat..

[195] Montano S.J., Grünler J., Nair D., Tekle M., Fernandes A.P., Hua X., Holmgren A., Brismar K., Ungerstedt J.S. (2015). Glutaredoxin mediated redox effects of coenzyme Q10 treatment in type 1 and type 2 diabetes patients. BBA Clin..

[196] Du Y., Zhang H., Montano S., Hegestam J., Ekberg N.R., Holmgren A., Brismar K., Ungerstedt J.S. (2014). Plasma glutaredoxin activity in healthy subjects and patients with abnormal glucose levels or overt type 2 diabetes. Acta Diabetol..

[197] Okuda M., Inoue N., Azumi H., Seno T., Sumi Y., Hirata K., Kawashima S., Hayashi Y., Itoh H., Yodoi J., Yokoyama M. (2001). Expression of glutaredoxin in human coronary arteries: its potential role in antioxidant protection against atherosclerosis. Arterioscler. Thromb. Vasc. Biol..

[198] Han J., Weisbrod R.M., Shao D., Watanabe Y., Yin X., Bachschmid M.M., Seta F., Janssen-Heininger Y.M.W., Matsui R., Zang M., Hamburg N.M., Cohen R.A. (2016). The redox mechanism for vascular barrier dysfunction associated with metabolic disorders: glutathionylation of Rac1 in endothelial cells. Redox Biol..

[199] Zimmet J.M., Hare J.M. (2006). Nitroso-redox interactions in the cardiovascular system. Circulation.

[200] Chen C.-A., Wang T.-Y., Varadharaj S., Reyes L.A., Hemann C., Talukder M.A.H., Chen Y.-R., Druhan L.J., Zweier J.L. (2010). S-glutathionylation uncouples eNOS and regulates its cellular and vascular function. Nature.

[201] Chen C.-A., De Pascali F., Basye A., Hemann C., Zweier J.L. (2013). Redox modulation of endothelial nitric oxide synthase by glutaredoxin-1 through reversible oxidative post-translational modification. Biochemistry.

[202] Karimi Galougahi K., Liu C.C., Garcia A., Gentile C., Fry N.A., Hamilton E.J., Hawkins C.L., Figtree G.A. (2016). β3 adrenergic stimulation restores nitric oxide/redox balance and enhances endothelial function in hyperglycemia. J. Am. Heart Assoc..

[203] Li C., Lv L., Li H., Yu D. (2012). Cardiac fibrosis and dysfunction in experimental diabetic cardiomyopathy are ameliorated by alpha-lipoic acid. Cardiovasc. Diabetol..

[204] Ho F.M., Lin W.W., Chen B.C., Chao C.M., Yang C.-R., Lin L.Y., Lai C.C., Liu S.H., Liau C.S. (2006). High glucose-induced apoptosis in human vascular endothelial cells is mediated through NF-kappaB and c-Jun NH2-terminal kinase pathway and prevented by PI3K/Akt/eNOS pathway. Cell. Signal..

[205] Wetzelberger K., Baba S.P., Thirunavukkarasu M., Ho Y.-S., Maulik N., Barski O.A., Conklin D.J., Bhatnagar A. (2010). Postischemic deactivation of cardiac aldose reductase: role of glutathione S-transferase P and glutaredoxin in regeneration of reduced thiols from sulfenic acids. J. Biol. Chem..

[206] Li Y., Hazarika S., Xie D., Pippen A.M., Kontos C.D., Annex B.H. (2007). In mice with type 2 diabetes, a vascular endothelial growth factor (VEGF)-activating transcription factor modulates VEGF signaling and induces therapeutic angiogenesis after hindlimb ischemia. Diabetes.

[207] Olsson A.-K., Dimberg A., Kreuger J., Claesson-Welsh L. (2006). VEGF receptor signalling - in control of vascular function. Nat. Rev. Mol. Cell Biol..

[208] Kendall R.L., Thomas K.A. (1993). Inhibition of vascular endothelial cell growth factor activity by an endogenously encoded soluble receptor. Proc. Natl. Acad. Sci. U. S. A..

[209] Hazarika S., Dokun A.O., Li Y., Popel A.S., Kontos C.D., Annex B.H. (2007). Impaired angiogenesis after hindlimb ischemia in type 2 diabetes mellitus: differential regulation of vascular endothelial growth factor receptor 1 and soluble vascular endothelial growth factor receptor 1. Circ. Res..

[210] Murdoch C.E., Shuler M., Haeussler D.J., Kikuchi R., Bearelly P., Han J., Watanabe Y., Fuster J.J., Walsh K., Ho Y.S., Bachschmid M.M., Cohen R.A., Matsui R. (2014). Glutaredoxin-1 up-regulation induces soluble vascular endothelial growth factor receptor 1, attenuating post-ischemia limb revascularization. J. Biol. Chem..

[211] Andreadou I., Efentakis P., Frenis K., Daiber A., Schulz R. (2021). Thiol-based redox-active proteins as cardioprotective therapeutic agents in cardiovascular diseases. Basic Res. Cardiol..

[212] Watanabe Y., Murdoch C.E., Sano S., Ido Y., Bachschmid M.M., Cohen R.A., Matsui R. (2016). Glutathione adducts induced by ischemia and deletion of glutaredoxin-1 stabilize HIF-1α and improve limb revascularization. Proc. Natl. Acad. Sci. U. S. A.

[213] Cohen R.A., Murdoch C.E., Watanabe Y., Bolotina V.M., Evangelista A.M., Haeussler D.J., Smith M.D., Mei Y., Tong X., Han J., Behring J.B., Bachschmid M.M., Matsui R. (2016). Endothelial cell redox regulation of ischemic angiogenesis. J. Cardiovasc. Pharmacol..

[214] Kropp M., Golubnitschaja O., Mazurakova A., Koklesova L., Sargheini N., Vo T.-T.K.S., de Clerck E., Polivka J., Potuznik P., Polivka J., Stetkarova I., Kubatka P., Thumann G. (2023). Diabetic retinopathy as the leading cause of blindness and early predictor of cascading complications-risks and mitigation. EPMA J..

[215] Kusuhara S., Fukushima Y., Ogura S., Inoue N., Uemura A. (2018). Pathophysiology of diabetic retinopathy: the old and the new. Diabetes Metab. J..

[216] Rübsam A., Parikh S., Fort P.E. (2018). Role of inflammation in diabetic retinopathy. Int. J. Mol. Sci..

[217] Feenstra D.J., Yego E.C., Mohr S. (2013). Modes of retinal cell death in diabetic retinopathy. J. Clin. Exp. Ophthalmol..

[218] Shin E.S., Sorenson C.M., Sheibani N. (2014). Diabetes and retinal vascular dysfunction. J. Ophthalmic Vis. Res..

[219] Stem M.S., Gardner T.W. (2013). Neurodegeneration in the pathogenesis of diabetic retinopathy: molecular mechanisms and therapeutic implications. Curr. Med. Chem..

[220] Wu F., Wang G.M., Raghavachari N., Lou M.F. (1998). Distribution of thioltransferase (glutaredoxin) in ocular tissues. Invest. Ophthalmol. Vis. Sci..

[221] Shelton M.D., Kern T.S., Mieyal J.J. (2007). Glutaredoxin regulates nuclear factor kappa-B and intercellular adhesion molecule in Müller cells: model of diabetic retinopathy. J. Biol. Chem..

[222] Shelton M.D., Distler A.M., Kern T.S., Mieyal J.J. (2009). Glutaredoxin regulates autocrine and paracrine proinflammatory responses in retinal glial (muller) cells. J. Biol. Chem..

[223] Qin D., Zhang G.-M., Xu X., Wang L.-Y. (2015). The PI3K/Akt signaling pathway mediates the high glucose-induced expression of extracellular matrix molecules in human retinal pigment epithelial cells. J. Diabetes Res..

[224] Karar J., Maity A. (2011). PI3K/AKT/mTOR pathway in angiogenesis. Front. Mol. Neurosci..

[225] Jiang N., Chen X.-L., Yang H.-W., Ma Y.-R. (2015). Effects of nuclear factor κB expression on retinal neovascularization and apoptosis in a diabetic retinopathy rat model. Int. J. Ophthalmol..

[226] Klein B.E., Klein R., Moss S.E. (1985). Prevalence of cataracts in a population-based study of persons with diabetes mellitus. Ophthalmology.

[227] Mulhern M.L., Madson C.J., Danford A., Ikesugi K., Kador P.F., Shinohara T. (2006). The unfolded protein response in lens epithelial cells from galactosemic rat lenses. Invest. Ophthalmol. Vis. Sci..

[228] Chan A.W.H., Ho Y., Chung S.K., Chung S.S.M. (2008). Synergistic effect of osmotic and oxidative stress in slow-developing cataract formation. Exp. Eye Res..

[229] Zhang J., Yan H., Lou M.F. (2017). Does oxidative stress play any role in diabetic cataract formation? –--Re-evaluation using a thioltransferase gene knockout mouse model. Exp. Eye Res..

[230] Löfgren S., Fernando M.R., Xing K.-Y., Wang Y., Kuszynski C.A., Ho Y.-S., Lou M.F. (2008). Effect of thioltransferase (glutaredoxin) deletion on cellular sensitivity to oxidative stress and cell proliferation in lens epithelial cells of thioltransferase knockout mouse. Invest. Ophthalmol. Vis. Sci..

[231] Ren X., Léveillard T. (2022). Modulating antioxidant systems as a therapeutic approach to retinal degeneration. Redox Biol..

[232] Parving H.H., Lewis J.B., Ravid M., Remuzzi G., Hunsicker L.G. (2006). Prevalence and risk factors for microalbuminuria in a referred cohort of type II diabetic patients: a global perspective. Kidney Int..

[233] Pambianco G., Costacou T., Ellis D., Becker D.J., Klein R., Orchard T.J. (2006). The 30-year natural history of type 1 diabetes complications: the pittsburgh epidemiology of diabetes complications study experience. Diabetes.

[234] Levin A., Nair D., Qureshi A.R., Bárány P., Heimburger O., Anderstam B., Stenvinkel P., Bruchfeld A., Ungerstedt J.S. (2018). Serum glutaredoxin activity as a marker of oxidative stress in chronic kidney disease: a pilot study. Nephron.

[235] Seghieri G., Di Simplicio P., De Giorgio L.A., Anichini R., Alberti L., Franconi F. (2000). Relationship between metabolic glycaemic control and platelet content of glutathione and its related enzymes, in insulin-dependent diabetes mellitus. Clin. Chim. Acta.

[236] Di Simplicio P., de Giorgio L.A., Cardaioli E., Lecis R., Miceli M., Rossi R., Anichini R., Mian M., Seghieri G., Franconi F. (1995). Glutathione, glutathione utilizing enzymes and thioltransferase in platelets of insulin-dependent diabetic patients: relation with platelet aggregation and with microangiopatic complications. Eur. J. Clin. Invest..

[237] Chen R., Shi J., Yin Q., Li X., Sheng Y., Han J., Zhuang P., Zhang Y. (2018). Morphological and pathological characteristics of brain in diabetic encephalopathy. J. Alzheimers Dis. JAD.

[238] Barbiellini Amidei C., Fayosse A., Dumurgier J., Machado-Fragua M.D., Tabak A.G., van Sloten T., Kivimäki M., Dugravot A., Sabia S., Singh-Manoux A. (2021). Association between age at diabetes onset and subsequent risk of dementia. JAMA.

[239] Yang K., Chen Z., Gao J., Shi W., Li L., Jiang S., Hu H., Liu Z., Xu D., Wu L. (2017). The key roles of GSK-3β in regulating mitochondrial activity, cell. Physiol. Biochem. Int. J. Exp. Cell. Physiol. Biochem. Pharmacol..

[240] Martin S.A., Souder D.C., Miller K.N., Clark J.P., Sagar A.K., Eliceiri K.W., Puglielli L., Beasley T.M., Anderson R.M. (2018). GSK3β regulates brain energy metabolism. Cell Rep..

[241] Engel T., Gómez-Sintes R., Alves M., Jimenez-Mateos E.M., Fernández-Nogales M., Sanz-Rodriguez A., Morgan J., Beamer E., Rodríguez-Matellán A., Dunleavy M., Sano T., Avila J., Medina M., Hernandez F., Lucas J.J., Henshall D.C. (2018). Bi-directional genetic modulation of GSK-3β exacerbates hippocampal neuropathology in experimental status epilepticus. Cell Death Dis..

[242] Qiu Z., Li X., Duan C., Li R., Han L. (2021). Glutaredoxin 1 protects neurons from oxygen-glucose deprivation/reoxygenation (OGD/R)-induced apoptosis and oxidative stress via the modulation of GSK-3β/Nrf2 signaling. J. Bioenerg. Biomembr..

[243] Lacerda D., Ortiz V., Türck P., Campos-Carraro C., Zimmer A., Teixeira R., Bianchi S., de Castro A.L., Schenkel P.C., Belló-Klein A., Bassani V.L., da Rosa Araujo A.S. (2018). Stilbenoid pterostilbene complexed with cyclodextrin preserves left ventricular function after myocardial infarction in rats: possible involvement of thiol proteins and modulation of phosphorylated GSK-3β. Free Radic. Res..

[244] Kothari V., Luo Y., Tornabene T., O’Neill A.M., Greene M.W., Geetha T., Babu J.R. (2017). High fat diet induces brain insulin resistance and cognitive impairment in mice. Biochim. Biophys. Acta, Mol. Basis Dis..

[245] Gannon O.J., Robison L.S., Salinero A.E., Abi-Ghanem C., Mansour F.M., Kelly R.D., Tyagi A., Brawley R.R., Ogg J.D., Zuloaga K.L. (2022). High-fat diet exacerbates cognitive decline in mouse models of Alzheimer’s disease and mixed dementia in a sex-dependent manner. J. Neuroinflammation.

[246] Reutzel M., Grewal R., Esselun C., Petry S.F., Linn T., Brandt A., Bergheim I., Eckert G.P. (2022). Effects of different standard and special diets on cognition and brain mitochondrial function in mice. Nutr. Neurosci..

[247] Akshintala D., Chugh R., Amer F., Cusi K. (2019). Nonalcoholic fatty liver disease: the overlooked complication of type 2 diabetes. Endotext.

[248] Bril F., Cusi K. (2016). Nonalcoholic fatty liver disease: the new complication of type 2 diabetes mellitus, endocrinol. Metab. Clin. North Am..

[249] Cusi K. (2020). A diabetologist’s perspective of non-alcoholic steatohepatitis (NASH): knowledge gaps and future directions. Liver Int. Off. J. Int. Assoc. Study Liver.

[250] Cusi K. (2020). Time to include nonalcoholic steatohepatitis in the management of patients with type 2 diabetes. Diabetes Care.

[251] Targher G., Bertolini L., Padovani R., Rodella S., Tessari R., Zenari L., Day C., Arcaro G. (2007). Prevalence of nonalcoholic fatty liver disease and its association with cardiovascular disease among type 2 diabetic patients. Diabetes Care.

[252] Targher G., Lonardo A., Byrne C.D. (2018). Nonalcoholic fatty liver disease and chronic vascular complications of diabetes mellitus. Nat. Rev. Endocrinol..

[253] Ahmad M.I., Umair Ijaz M., Hussain M., Ali Khan I., Mehmood N., Siddiqi S.M., Liu C., Zhao D., Xu X., Zhou G., Li C. (2020). High fat diet incorporated with meat proteins changes biomarkers of lipid metabolism, antioxidant activities, and the serum metabolomic profile in Glrx1(-/-) mice. Food Funct..

[254] Ahmad M.I., Zou X., Ijaz M.U., Hussain M., Liu C., Xu X., Zhou G., Li C., Meat Protein Promoted Inflammation Processed, Lipogenesis Hepatic (2019). By upregulating Nrf2/Keap1 signaling pathway in glrx-deficient mice. J. Agric. Food Chem..

[255] Kosmalski M., Śliwińska A., Drzewoski J. (2023). Non-alcoholic fatty liver disease or type 2 diabetes mellitus-the chicken or the egg dilemma. Biomedicines.

[256] Burns M., Rizvi S.H.M., Tsukahara Y., Pimentel D.R., Luptak I., Hamburg N.M., Matsui R., Bachschmid M.M. (2020). Role of glutaredoxin-1 and glutathionylation in cardiovascular diseases. Int. J. Mol. Sci..

[257] Nishikawa T., Edelstein D., Du X.L., Yamagishi S., Matsumura T., Kaneda Y., Yorek M.A., Beebe D., Oates P.J., Hammes H.P., Giardino I., Brownlee M. (2000). Normalizing mitochondrial superoxide production blocks three pathways of hyperglycaemic damage. Nature.

[258] Elsner M., Gehrmann W., Lenzen S. (2011). Peroxisome-generated hydrogen peroxide as important mediator of lipotoxicity in insulin-producing cells. Diabetes.

[259] Newsholme P., Morgan D., Rebelato E., Oliveira-Emilio H.C., Procopio J., Curi R., Carpinelli A. (2009). Insights into the critical role of NADPH oxidase(s) in the normal and dysregulated pancreatic beta cell. Diabetologia.

[260] Hauck A.K., Bernlohr D.A. (2016). Oxidative stress and lipotoxicity. J. Lipid Res..

[261] Davì G., Falco A., Patrono C. (2005). Lipid peroxidation in diabetes mellitus. Antioxidants Redox Signal..

[262] Mertoglu C., Siranli G., Coban T.A., Karakurt Y., Ersoy A., Ozcicek A., Arslan Y., Gok G., Erel O. (2021). The role of protein oxidation in the development of diabetic microvascular complications. North. Clin. Istanb..

[263] Huang J., Jones D., Luo B., Sanderson M., Soto J., Abel E.D., Cooksey R.C., McClain D.A. (2011). Iron overload and diabetes risk: a shift from glucose to Fatty Acid oxidation and increased hepatic glucose production in a mouse model of hereditary hemochromatosis. Diabetes.

[264] Cooksey R.C., Jouihan H.A., Ajioka R.S., Hazel M.W., Jones D.L., Kushner J.P., McClain D.A. (2004). Oxidative stress, beta-cell apoptosis, and decreased insulin secretory capacity in mouse models of hemochromatosis. Endocrinology.

[265] Hanschmann E.-M., Petry S.F., Eitner S., Maresch C.C., Lingwal N., Lillig C.H., Linn T. (2020). Paracrine regulation and improvement of β-cell function by thioredoxin. Redox Biol..

